# Loneliness is linked to specific subregional alterations in hippocampus-default network covariation

**DOI:** 10.1152/jn.00339.2021

**Published:** 2021-11-24

**Authors:** Chris Zajner, R. Nathan Spreng, Danilo Bzdok

**Affiliations:** ^1^McConnell Brain Imaging Centre (BIC), Montreal Neurological Institute (MNI), Faculty of Medicine, McGill University, Montreal, Quebec, Canada; ^2^Department of Neurology and Neurosurgery, McGill University, Montreal, Quebec, Canada; ^3^Departments of Psychiatry and Psychology, McGill University, Montreal, QC, Canada; ^4^Douglas Mental Health University Institute, Verdun, Quebec, Canada; ^5^Department of Biomedical Engineering, Faculty of Medicine, McGill University, Montreal, Quebec, Canada; ^6^Mila—Quebec Artificial Intelligence Institute, Montreal, Quebec, Canada

**Keywords:** default network fragmentation, higher-order association cortex, hippocampus subfields, polygenic risk score prediction, population neuroscience

## Abstract

Social interaction complexity makes humans unique. But in times of social deprivation, this strength risks exposure of important vulnerabilities. Human social neuroscience studies have placed a premium on the default network (DN). In contrast, hippocampus (HC) subfields have been intensely studied in rodents and monkeys. To bridge these two literatures, we here quantified how DN subregions systematically covary with specific HC subfields in the context of subjective social isolation (i.e., loneliness). By codecomposition using structural brain scans of ∼40,000 UK Biobank participants, loneliness was specially linked to midline subregions in the uncovered DN patterns. These association cortex patterns coincided with concomitant HC patterns implicating especially CA1 and molecular layer. These patterns also showed a strong affiliation with the fornix white matter tract and the nucleus accumbens. In addition, separable signatures of structural HC-DN covariation had distinct associations with the genetic predisposition for loneliness at the population level.

**NEW & NOTEWORTHY** The hippocampus and default network have been implicated in rich social interaction. Yet, these allocortical and neocortical neural systems have been interrogated in mostly separate literatures. Here, we conjointly investigate the hippocampus and default network at a subregion level, by capitalizing structural brain scans from ∼40,000 participants. We thus reveal unique insights on the nature of the “lonely brain” by estimating the regimes of covariation between the hippocampus and default network at population scale.

## Graphical abstract

**Figure:**
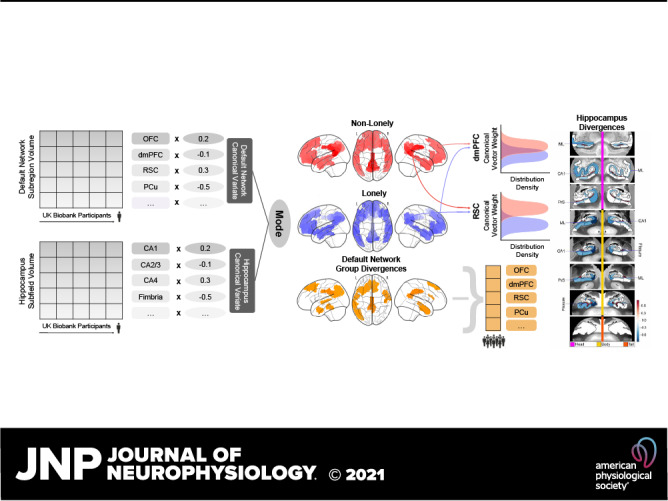

## INTRODUCTION

Social isolation can be detrimental. Accumulating evidence suggests social disconnection as a major risk factor for morbidity and mortality (1, 2). Indeed, it has been found that ∼10–20% of adults who live alone report feeling lonely (3). Yet, it is becoming increasingly clear that experiencing feelings of social isolation, namely loneliness, has diverging biological correlates compared with an objective lack of social contact with others (4). Conversely, individuals who are well surrounded by others can still experience a feeling of inadequate social connection (5). Therefore, it is widely acknowledged that there can be a divide between subjective satisfaction with their social relationships and people’s objective level of social support.

Lonely individuals are particularly characterized by consistent cognitive biases related to processing social cues in the environment. Additionally, loneliness was reported to entail a state of hypervigilance for threats in one’s social environment (5). According to some authors, an aversive feeling of loneliness serves as a biological warning signal that alerts individuals to improve their social relationships (6). Yet, the impact of loneliness extends well beyond the realm of social interaction. For example, previous studies showed that lonely individuals tend to prioritize nonsocial rewards over social rewards (7) and have reduced cognitive control (8), reduced immune response to viruses (6, 9), heightened stress response (10, 11), poorer mental health (1), increased susceptibility to major psychiatric disorders (1), as well as higher risks for alcohol abuse (12), committing suicide (1, 3), cardiovascular disease (13, 14), cognitive decline, and Alzheimer’s disease (15–17). The association with Alzheimer’s disease also motivated our investigation of the hippocampus (HC) and default network (DN) because the hippocampus (18) and DN (19) have long been recognized as a primary neural pathway implicated in the pathophysiology of this major neurodegenerative disease.

Furthermore, while disruption of memory capacity is one of the hallmarks of Alzheimer’s disease (20), the DN and HC have both been implicated in environment-independent processing. This invokes cognitive processes such as episodic memory and mental scene construction, which also show alterations in lonely individuals (21–23). That these two neural systems should relate to loneliness may thus be unsurprising. Indeed, past literature on loneliness has insisted on the importance of rumination on self-focused memories (24), poor executive control (8, 25), and negative bias in the perception of social cues (cf. above). In fact, the aspects of loneliness that rely upon internally generated dimensions of cognition have been argued to especially relate to the DN (26). In rodents and monkeys, the HC has been found to support highly synchronized and spontaneous neural activity bursts during states of rest, so-called sharp-wave ripples (SWRs). These rapid electrophysiological bursts originate in specific anatomical areas of the hippocampus, such as CA1, known to be closely affiliated with DN regions, especially with the medial prefrontal cortex (mPFC) (27). For example, in monkey brains, hippocampal CA1 neurons have been shown to send direct axonal output connections to the mPFC through the fornix white matter pathway (28). This fact could in part explain the recent finding that the fornix is the major white matter tract that is most strongly linked to loneliness (29). The nucleus accumbens (NAc), which is another major target site of hippocampal CA1 neurons and the fornix (28, 30), has likewise been linked to the craving for social connection (31). Alteration of the NAc in loneliness, and thus the reward circuitry of the brain, may therefore be a product of this common anatomical pathway. In light of these details, it has been concluded that since “the pyramidal neurons of the CA1 region provide the only hippocampal output to cortical targets, this activity must have functional significance. We just have to figure out what it is” (32). To take a few steps in this direction, our study aimed to zoom in on the HC-DN correspondence in the context of subjective social isolation.

Indeed, recent evidence has shown that DN gray matter (GM) morphology is strongly associated with loneliness (29). Analogously, the hippocampus has been discussed to be particularly affected by social isolation in various animal species (33–36). Moreover, researchers have been able to perform these invasive studies on the animal hippocampus at a subregion and even single-cell level. Such fine-grained resolution has been difficult to achieve with respect to DN subregions in the living human brain. Since the HC and DN subregions are closely related, studying their structural divergence in lonely individuals provides a window that can offer refined understanding of the association between loneliness and its brain basis. By joint analysis of the HC and DN, the existing in-depth knowledge of the animal HC may help elucidate the nature of potentially human-specific DN subregions. Moreover, in addition to high-resolution structural brain imaging and social isolation information, the availability of genetic data allowed us to also investigate the differing contributions of genetic influences on structural brain patterns related to loneliness.

Previous brain imaging studies aimed at brain parcellation have typically investigated either the default network alone (e.g., Ref. 37) or the hippocampus alone (e.g., Ref. 38). Additionally, there is still insufficient work relating animal studies on anatomically defined subregions of the hippocampus to what can be reliably measured in the human medial temporal lobe with brain imaging techniques. Advances in the automatic segmentation of the hippocampus using ex vivo brain imaging (39, 40) now allow the reliable assessments of microanatomically defined hippocampus subregions in a way that scales to the ∼40,000 UK Biobank Imaging cohort. This enables deeper analyses of the principled interrelationships between the evolutionarily more conserved hippocampus of the allocortex and the default network of the recently expanded neocortex. By leveraging a framework for high-dimensional codecomposition at a fine-grained subregion resolution, we here investigate the structural deviations of the HC-DN covariation signatures that characterize loneliness.

## MATERIALS AND METHODS

### Population Data Resource

The UK Biobank is a prospective epidemiology resource that offers extensive behavioral and demographic assessments, medical and cognitive measures, as well as biological samples in a cohort of ∼500,000 participants recruited from across Great Britain (https://www.ukbiobank.ac.uk/). This openly accessible population data set aims to provide brain imaging for ∼100,000 individuals, planned for completion in 2022. The present study was based on the recent data release from February/March 2020. To improve comparability and reproducibility, our study built on the uniform data preprocessing pipelines designed and carried out by FMRIB, Oxford University, Oxford, UK (41). Our study involved data from 38,701 participants with brain imaging measures and expert-curated image-derived phenotypes of gray matter morphology (T1-weighted MRI) from 48% men and 52% women, aged 40–69 yr when recruited [mean age 55 yr, standard deviation (SD) 7.5 yr]. The present analyses were conducted under UK Biobank application number 25163. All participants provided written informed consent. Further information on the consent procedure can be found elsewhere (http://biobank.ctsu.ox.ac.uk/crystal/field.cgi?id=200).

### Loneliness Target Phenotype

Regarding the loneliness target phenotype, we used the yes/no answer from UK Biobank participants to the question “Do you often feel lonely?” (data field 2020) as a subjective indicator of the quality of social interactions. Measures of social relationship quality represent a widely accepted and commonly investigated component of social embeddedness (42, 43). Loneliness is more commonly viewed as a subjective feeling of being alone, regardless of social encounter frequency (44). Conceptually similar and highly correlated scales (45) are also contained in other standard measurement tools of social embeddedness, such as the Revised UCLA Loneliness Scale (43) and the Interpersonal Support Evaluation List (42). In general, a variety of studies showed single-item measures of social isolation traits to be reliable and valid (46–48). The demographic differences between lonely and nonlonely individuals in the UK Biobank have been reported elsewhere (29).

### Brain Imaging and Preprocessing Procedures

Magnetic resonance imaging scanners (3-T Siemens Skyra) were matched at several dedicated data collection sites with the same acquisition protocols and standard Siemens 32-channel radiofrequency receiver head coils. To protect the anonymity of the study participants, brain imaging data were defaced and any sensitive metainformation was removed. Automated processing and quality control pipelines were deployed (41, 49). To improve homogeneity of the imaging data, noise was removed by means of 190 sensitivity features. This approach allowed for the reliable identification and exclusion of problematic brain scans, such as due to excessive head motion.

The structural MRI data were acquired as high-resolution T1-weighted images of brain anatomy with a three-dimensional (3-D) magnetization-prepared rapid gradient echo (MPRAGE) sequence at 1-mm isotropic resolution. Preprocessing included gradient distortion correction (GDC), field of view reduction using the Brain Extraction Tool (50), and FLIRT (51, 52), as well as nonlinear registration to MNI152 standard space at 1-mm resolution using FNIRT (53). To avoid unnecessary interpolation, all image transformations were estimated, combined, and applied by a single interpolation step. Tissue type segmentation into cerebrospinal fluid, gray matter, and white matter was applied by using FAST [FMRIB’s Automated Segmentation Tool (54)] to generate full bias-field-corrected images. SIENAX (55), in turn, was used to derive volumetric measures normalized for head size.

For the default network, volume extraction was anatomically guided by the Schaefer–Yeo reference atlas (56). Among the total of 400 parcels, 91 subregion definitions are provided as belonging to the default network among the seven canonical networks. For the hippocampus, 38 volume measures were extracted with the automatic FreeSurfer subsegmentation (40). The allocortical volumetric segmentation draws on a probabilistic hippocampus atlas with ultrahigh resolution at ∼0.1 mm isotropic. In particular, this tool from the FreeSurfer 7.0 suite gives special attention to surrounding anatomical structures to refine the hippocampus subregion segmentation in each participant.

As a preliminary data-cleaning step, building on previous UK Biobank research (29, 57), interindividual variations in brain region volumes that could be explained by nuisance variables of no interest were regressed out: body mass index, head size, head motion during task-related brain scans, head motion during task-unrelated brain scans, head position and receiver coil in the scanner (*x*, *y*, and *z*), position of scanner table, as well as the data acquisition site, in addition to age, age^2^, sex, sex × age, and sex × age^2^. The cleaned volumetric measures from the 91 DN subregions in the neocortex and the 38 HC subregions in the allocortex served as the basis for all subsequent analysis steps.

### Structural Variation Relationships of the Fornix and Nucleus Accumbens

In the context of HC-DN correspondence, the fornix white matter tract plays a central role for several important reasons. The fornix constitutes the major output pathway of the hippocampus, carrying information unidirectionally toward the neocortical mantle (28). Our previous research has shown that the fornix is the major white matter tract whose microstructural variation is by far most predictable based on DN subregion volumes alone (58). This fiber pathway has also recently been shown to be linked to loneliness (29). Additionally, on its way, the fornix carries axons from hippocampus neurons to the nucleus accumbens (28, 30)—a core node of the reward circuitry, which has been implicated in the sequelae of loneliness, such as substance abuse (2).

For these reasons, we have conducted preliminary regression analyses for the fornix [UK Biobank (UKB) data fields 25095, 25094, and 25061] and for the nucleus accumbens (UKB data fields 25024 and 25023). The ancillary analyses explored their structural relationships with the subregions composing our DN atlas and those composing our HC atlas (cf. above). The model specification for these regression analyses was as follows:

$$y=x_{1}\;\times \;\beta_{region_{1}}\;+\;\ldots \;+\;x_{j}\;\times \;\beta_{region_{j}}$$

where β denotes the slope parameters corresponding to the brain volumes **x** for all subregion volumes of either the DN atlas or the HC atlas (*z* scored across participants) and *y* denotes the mean fractional anisotropy (FA) of the fornix or the volume of the nucleus accumbens (*z* scored across participants) for the UK Biobank participants.

### Analysis of Covariation between Hippocampus Subregions and Default Network Subregions

As the central step of the analytical workflow, we sought dominant regimes of structural correspondence—signatures or “modes” of population covariation that provide insights into how structural variation among the segregated HC can explain structural variation among the segregated DN. Canonical correlation analysis (CCA) was a natural choice of method to interrogate such a multivariate interrelation between two high-dimensional variable sets (59–61). A first variable set *X* was constructed from the DN subregion volumes (number of participants × 91 DN parcels matrix). A second variable set *Y* was constructed from the HC subregion volumes (number of participants × 38 HC parcels matrix):

$$X\in {\mathbb{R}}^{n\times p}$$

$$Y\in {\mathbb{R}}^{n\times q}$$

where *n* denotes the number of observations or participants, *p* is the number of DN subregions, and *q* is the number of HC subregions. Each column of the two data matrices was *z* scored to zero mean (i.e., centering) and unit variance (i.e., rescaling). CCA addresses the problem of maximizing the linear correlation between low-rank projections from the two variable sets or data matrices. The two sets of linear combinations of the original variables are obtained as follows:

$$L_{X}=XV\;\;\;L_{Y}=YU$$

$$\begin{array}{cc} l_{X,l}=Xv_{l} & l_{Y,l}=Yu_{l} \end{array}$$

$$\mathrm{corr}\left(l_{X,l},l_{Y,l}\right)\propto l_{X,l}^{T}l_{Y,l}=\max$$

where *V* and *U* denote the respective contributions of *X* and *Y*, *L_X_* and *L_Y_* denote the respective latent “modes” of joint variation based on patterns derived from *X* and patterns derived from *Y*, *l_X,l_* is the *l*th column of *L_X_*, and *l_Y,l_* is the *l*th column of *L_Y_*. We define modes as general principles of population variation in our target neural circuits that can be reliably extracted in brain structure at the population level. The goal of our CCA application was to find pairs of latent vectors *l_X,l_* and *l_Y,l_* with maximal correlation in the derived latent embedding. In an iterative process, the data matrices *X* and *Y* were decomposed into *L* components, where *L* denotes the number of modes given the model specification. In other words, CCA involves finding the canonical vectors *u* and *v* that maximize the (symmetric) relationship between a linear combination of DN volume expressions (*X*) and a linear combination of HC volume expressions (*Y*). CCA thus identifies the two concomitant projections *Xv_l_* and *Yu_l_*. These yielded the optimized co-occurrence between patterns of subregion variation inside the segregated DN and patterns of subregion variation inside the segregated HC across participants.

In other words, each estimated cross-correlation signature identified a constellation of within-DN volumetric variation and a constellation of within-HC volumetric variation that go hand in hand with each other. The set of *k* orthogonal modes of population covariation are mutually uncorrelated by construction (60). They are also naturally ordered from the most important to the least important HC-DN covariation mode based on the amount of variance explained between the allocortical and neocortical variable sets. The first and strongest mode therefore explained the largest fraction of joint variation between combinations of HC subregions and combinations of DN subregions. Each ensuing cross-correlation signature captured a fraction of structural variation that is not explained by one of the *k* − 1 other modes. The variable sets were entered into CCA after a confound-removal procedure based on previous UK Biobank research (cf. above).

### Group Difference Analysis

For the derived population modes of HC-DN covariation, we then performed a rigorous group contrast analysis in the context of loneliness. We aimed to identify which anatomical subregions show statistically defensible deviation in the lonely group compared with the control group. We carried out a principled test for whether the solution vector obtained from the CCA (i.e., canonical vectors, cf. above) in the lonely group is systematically different from the solution vector in the control group.

More specifically, following previous UK Biobank research (57), we carried out a bootstrap difference test of the CCA solution from the lonely versus nonlonely groups (62). In 100 bootstrap iterations, we randomly pulled participant samples with replacement to build an alternative data set (with the same sample size) that we could have gotten. We subsequently performed CCA in parallel by fitting one separate model to each of the two groups. In each resampling iteration, this approach thus carried out a separate estimation of the doubly multivariate correspondence between HC subregions and DN subregions in each particular group. The two distinct CCA solutions from each iteration were then matched mode by mode regarding sign invariance and mode order. Canonical vectors of a given mode that carried opposite signs were aligned by multiplying one with −1. The order of the CCA modes was aligned based on pairwise Pearson’s correlation coefficient between the canonical vectors from each estimated CCA model. After mode matching, we directly estimated the resample-to-resample effects of model parameter estimates by elementwise subtraction of the corresponding canonical vectors of a given mode *k* between the two groups. We finally recorded these difference estimates for each vector entry (each corresponding to the degree of deviation in 1 particular anatomical subregion). The subregion-wise differences were ultimately aggregated across the 100 bootstrap data sets to obtain a nonparametric distribution of group contrast estimates.

We thus propagated the variability attributable to participant sampling into the computed uncertainty estimates of group differences in the UK Biobank population cohort. Statistically relevant alterations of anatomical subregions in loneliness were determined by whether the two-sided confidence interval included zero or not according to the 10/90% bootstrap-derived distribution of difference estimates (57) in an approach that is faithful to our multivariate analytical strategy and research question at hand. This nonparametric approach directly quantified the statistical uncertainty of how loneliness is manifested in specific subregions of the HC-DN axis.

### Analysis of How Individual Expressions of Hippocampus-DN Covariation Are Linked to the Genetic Predisposition for Loneliness

Polygenic risk score (PRS) is a genome-wide analysis technique that has been shown to successfully quantify individuals’ genetic predisposition for a variety of phenotypes. The approach has become especially potent for complex phenotypes that implicate tens of thousands of single-nucleotide polymorphisms (SNPs) with individually small effect sizes, such as major psychiatric diseases (63–67). PRSs have also come to be a sharp tool for heritability analyses because of the advent of large population data sets such as the UK Biobank (68, 69). Such data resources have allowed the investigation of the relationship between SNP variation and interindividual differences in a particular phenotype, which includes neuroimaging-derived phenotypes (70, 71). For the purpose of the present study, we have constructed PRS models for the loneliness trait. The subject-specific risk scores were then regressed onto our expressions of HC-DN modes (i.e., canonical variates). The integrative imaging-genetics approach allowed disentangling which mode expressions showed reliable relationships to the heritability of loneliness as attributable to thousands of genetic variants.

As is common for PRS analysis workflows, summary statistics from previously conducted genome-wide association studies (GWASs) on our target phenotypes were used as the backdrop to determine how several hundred thousand SNPs are associated with the loneliness trait. The summary statistics for loneliness were obtained from a GWAS that was conducted as part of the Psychiatric Genomics Consortium. Quality control was implemented by excluding SNPs with a minor allele frequency of <1%, as well as excluding SNPs with imputation information score of <0.8. Mismatching, duplicate, and ambiguous SNPs were also disregarded from further analysis. Quality control on the base data also involved excluding individuals with a difference between reported sex and that indicated by their sex chromosomes and removing overlapping samples.

The quality-controlled summary statistics were used as starting point for the PRS model that was built and applied with the PRSice framework (http://www.prsice.info). This software tool uses the available collection of effect sizes of candidate SNPs to form single-subject predictions of genetic predisposition for a phenotype of interest. In particular, this tool determined the optimal PRS model based on the UK Biobank participants (training data, *n* = 253,295) of European ancestry who did not provide any brain imaging data (at the time of study). This model training step involved automated adjustments, such as identifying ideal clumping and pruning choices, to select the thresholds that decide which SNPs are included in the PRS model. Subsequently, once optimized, the final PRS model was then used to predict the genetic predisposition for each of 23,423 UK Biobank participants of European ancestry with brain imaging data (test data). This PRS model consisted of the additive effects of weighted SNPs, whereby the weighted sum of the participants’ genotypes was computed as follows:

$$prs_{j}=\sum g_{\mathrm{ij}}\times {\hat{\text{β}}}_{i}\;$$

where g*_i_* denotes an individual’s genotype at SNP *i* (value 0, 1, or 2), ${\;\hat{\text{β}}}_{i}$ is the obtained point estimates of the per-allele effect sizes at SNP *i*, and *j* is a particular individual (68).

Finally, Bayesian linear regression was used to regress the subject-specific predictions of genetic liability for the loneliness trait onto the participant expressions of HC-DN covariation modes. More specifically, the individuals in the top 5% predictions (i.e., highest PRS estimates) and the individuals in the bottom 5% predictions (i.e., lowest PRS estimates) were considered in a Bayesian logistic regression model with mode expressions serving as input variables (72–74). In this multiple regression setup, PRS was regressed against each of the 25 canonical variates (linearly uncorrelated by construction) for each individual on the hippocampus side. An analogous multiple regression model was estimated for the (uncorrelated) 25 canonical variates from the DN side. The fully Bayesian model specification for these regression analyses was as follows:

$$y_{\mathrm{prs}}=x_{1}\;\times \;\beta_{mode_{1}}\;+\;\ldots \;+\;x_{25}\;\times \;\beta_{mode_{25}}+\alpha_{\mathrm{men}\left[\mathrm{sex}\right]}+\;\alpha_{\mathrm{women}\left[\mathrm{sex}\right]}+\;\alpha_{\mathrm{men}\_\mathrm{age}\left[\mathrm{sex}\right]}\;\times \;\mathrm{ag}e_{\mathrm{men}}\;+\;\alpha_{\mathrm{women}\_\mathrm{age}\left[\mathrm{sex}\right]}\;\times \;\mathrm{ag}e_{\mathrm{women}}$$

$$\;\beta_{j}\;∼\;N_{j}\left(0,\;1\right)$$

$$\alpha_{\mathrm{men}}\;∼\;N\left(0,\;1\right)$$

$$\alpha_{\mathrm{women}}\;∼\;N\left(0,\;1\right)$$

$$\alpha_{\mathrm{men}\_\mathrm{age}}\;∼\;N\left(0,\;1\right)$$

$$\alpha_{\mathrm{women}\_\mathrm{age}}\;∼\;N\left(0,\;1\right)$$

where β*_j_* denote the slopes for the subject-specific 25 mode expressions as canonical variates *x_j_*; *y*_prs_ denotes the PRS estimates of each participant. Potential confounding influences were acknowledged by the nuisance variables α, which accounted for variation that could be explained by sex and (*z* scored) age. Once the Bayesian model solution was approximated with Markov chain Monte Carlo sampling, it yielded fully probabilistically specified posterior parameter distributions for each β coefficient corresponding to one of the signatures of allocortical-neocortical covariation (cf. above). The association with trait heritability of a mode expression was then determined based on how robustly their corresponding model coefficients deviated from 0 (e.g., >95% of model coefficient posterior probability excluded a value of 0) (173, 174).

## RESULTS

Previous brain imaging studies aimed at brain parcellation have typically investigated either the default network alone (e.g., Ref. 37) or the hippocampus alone (e.g., Ref. 38). Additionally, there is still insufficient work relating animal studies on anatomically defined subregions of the hippocampus to what can be reliably measured in the human medial temporal lobe with brain imaging techniques. Advances in the automatic segmentation of the hippocampus using ex vivo brain imaging (39, 40) now allow the reliable assessments of microanatomically defined hippocampus subregions in a way that scales to the ∼40,000 UK Biobank Imaging cohort. This enables deeper analyses of the principled interrelationships between the evolutionarily more conserved hippocampus of the allocortex and the default network of the recently expanded neocortex. By leveraging a framework for high-dimensional codecomposition at a fine-grained subregion resolution, we here investigate the structural deviations of the HC-DN covariation signatures that characterize loneliness.

### Structural Variation Relationships of the Fornix

**Figure 1.**
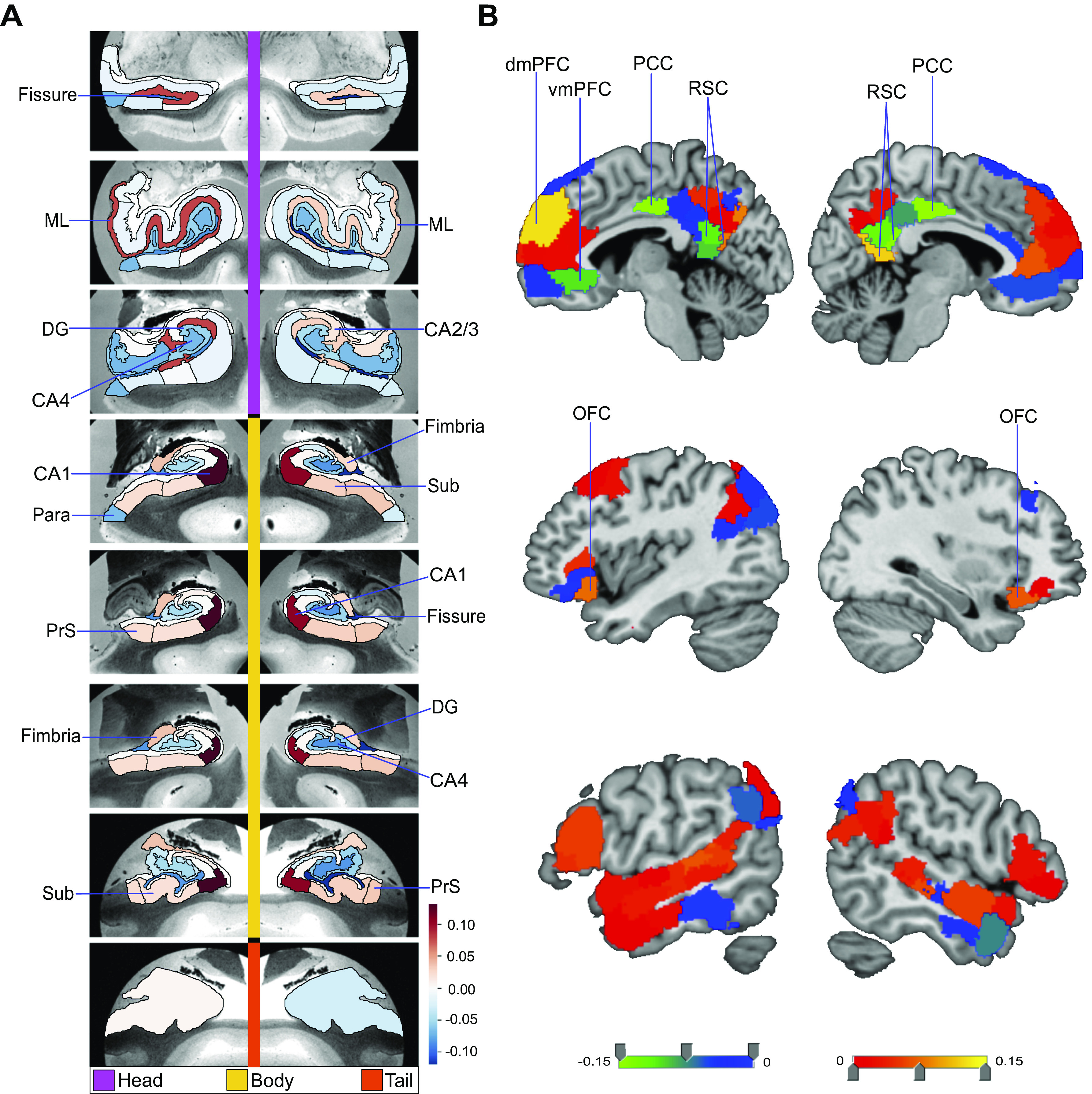
Structural correlates of the fornix white matter tract. How can microstructural variation of the fornix be explained as a function of subregion volume variations in the hippocampus (*left*) or the default network (DN) (*right*)? To address these two questions, we have computed separate exploratory multiple regression analyses based on 38,701 UK Biobank participants. The regression parameter weights corresponding to each specific subregion (hot and cold colors) outline the structural associations with fornix integrity exclusively explained by that particular subregion. *A* shows the hippocampus-fornix model based on 38 subregions from the hippocampus segmentation (40). The parameter weights indicate the variation explained in the fornix specifically by each hippocampal subregion, mapped onto 8 consecutive coronal sections of the left and right hippocampus from anterior (*top*) to posterior (*bottom*) (hot/cold colors = positive/negative volume association). The subregions with the strongest positive volume effect are CA1 body of the left and right hippocampus as well as left molecular layer head. The strongest negative relations with fornix microstructure are found in left and right hippocampal fissures. *B* shows the DN-fornix model based on 91 subregions in the Schaefer–Yeo parcellation of the DN. The parameter weights indicate the fornix variation explained specifically by each DN subregion. The subregions with the strongest marginal volume effect among DN subregions are bilateral posterior cingulate cortex (PCC), bilateral retrosplenial cortex (RSC), and left dorsal-medial prefrontal cortex (dmPFC). Overall, these results suggest that fornix architecture has differentiated structural relationships with subregions of the hippocampus and midline DN. DG, granule cell layer of dentate gyrus (GC-DG-ML); ML, molecular layer; OFC, orbitofrontal cortex; Para, parasubiculum; PrS, presubiculum; Sub, subiculum, vmPFC, ventromedial prefrontal cortex.

In a preliminary set of exploratory analyses, we sought to elucidate whether individual subregion volumes of the hippocampus could distinctly explain microstructural variation of the fornix white matter tract—an important fiber pathway known to carry axons from the allocortical hippocampus to the DN. To this end, we performed a multiple regression analysis to regress the structural integrity of the fornix (mean FA, diffusion MRI) onto 38 hippocampal subregion volumes. We found that the bilateral CA1 body dominates in explaining variation in fornix integrity, followed by the molecular layer (ML) head of the left hippocampus (Fig. 1*A*). Smaller positive effects were found in the bilateral subiculum body, bilateral presubiculum body, bilateral fimbria, right ML head, and right CA2/3. We found negative effects in the bilateral hippocampal fissure, left parasubiculum, bilateral CA4 head, as well as bilateral CA4 body. These findings indicate that several hippocampal subregions have unique structural relationships with fornix integrity. In a variant of this analysis, rather than marginal or partial association strengths we computed pairwise Pearson correlation strengths between fornix integrity and each hippocampal subregion. The hippocampus subregions with the largest correlation coefficient rho were also the subregions with the greatest amount of explained variance in the multiple regression analysis (Supplemental Fig. S1; all Supplemental Material can be found at https://doi.org/10.6084/m9.figshare.15060684). Thus, from convergent evidence across two different analyses of how hippocampus subregions track fornix variation, the CA1 body showed the strongest positive association with the fornix, whereas the hippocampal fissure displayed the strongest negative association.

We next sought to regress variation of fornix integrity onto 91 subregion volumes of the DN (Fig. 1*B*). The subregions with the strongest positive effects included the left dorsal mPFC (dmPFC) and the right retrosplenial cortex (RSC). There was also a small effect in the left RSC, bilateral ventromedial prefrontal cortex (vmPFC), and left dorsolateral prefrontal cortex (dlPFC). Conversely, we located the strongest negative effects to the bilateral posterior cingulate cortex (PCC). Furthermore, in the companion analysis using Pearson correlation between each DN subregion and the fornix, mPFC subregions had in general strong positive effects (Supplemental Fig. S2). On the other hand, PCC/RSC subregions had weak or negative effects (Supplemental Fig. S2). These findings thus made apparent the subregion-specific relationships of how DN subregions track fornix integrity in our UKB cohort, with lateral DN subregions showing more subtle effects and midline DN subregions showing the most prominent effects.

### Structural Variation Relationships of the Nucleus Accumbens

Using the identical analysis approach (cf. above), we next explored how the volume of the nucleus accumbens (NAc) can be explained as a function of hippocampal subregion volumes. These multiple regression analyses revealed that bilateral CA1 body and right molecular layer head subregions explained the most variation in NAc volume (Supplemental Fig. S2A). Small positive effects were also found in CA2/3 head of the right hippocampus, left molecular layer head, and bilateral hippocampus amygdala transition area (HATA). Conversely, there was a strong negative association of the NAc with bilateral hippocampal fissure as well as with left parasubiculum. Interrogating the HC-NAc relationships thus showed distinct explanatory effects among subregions. In particular, the hippocampal subregions that explained the most variation in NAc recapitulated those that also best explained fornix integrity (cf. above). To further investigate the associations of the NAc, we next computed gross pairwise Pearson correlation coefficients of each hippocampal subregion with the NAc. We observed a noticeably stronger correlation for CA1 head than any other subregion (Supplemental Fig. S3), although CA1 head showed a negligible effect in the multiple regression analysis of the NAc on hippocampus subregions. Thus, although CA1 head on its own has a strong correlation with the NAc, when considered in a joint model with all other hippocampal subregions in our atlas it explained little NAc variation in a unique way. This observation suggests that distinct hippocampal subregions, other than CA1, make separate contributions that together better explain volume variation in the NAc.

**Figure 2.**
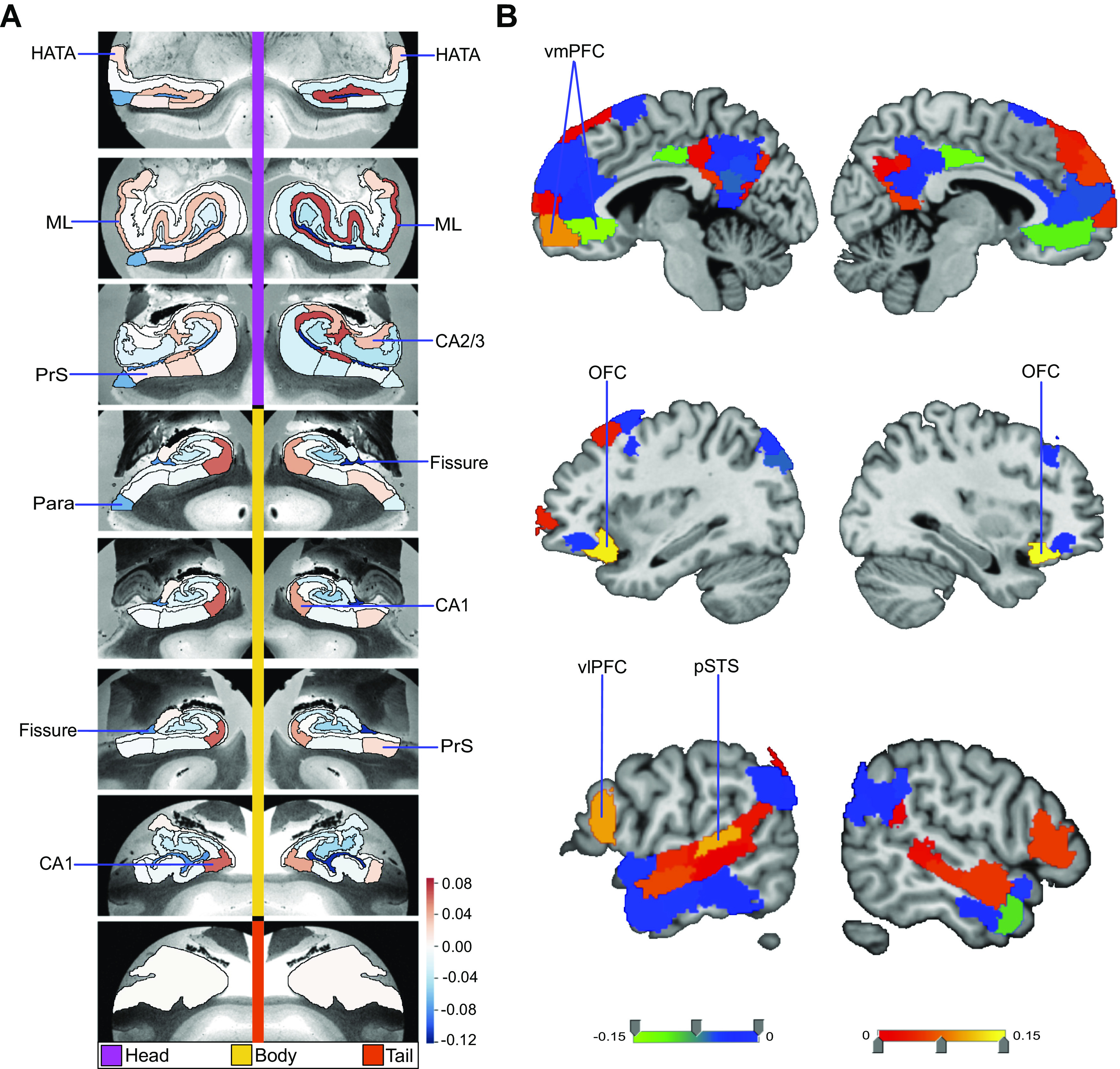
Structural correlates of the nucleus accumbens (NAc). Regression parameter weights of various subregions in separate exploratory multiple regression analyses outline the volume effects on the NAc, due exclusively to each subregion in either the hippocampus or default network. *A* shows the hippocampus (HC)-NAc model mapped onto 8 consecutive coronal slices of the left and right HC in the anterior (*top*)-to-posterior (*bottom*) direction. The parameter weights indicate the structural variation uniquely explained in the NAc specifically by each hippocampal subregion (hot/cold colors = positive/negative volume association). The subregions with the strongest positive volume effect are CA1 body of the left and right hippocampus as well as right molecular layer (ML) head, in line with our analogous analyses on the fornix (Fig. 1*A*). The strongest negative volume effects are left and right hippocampal fissures. *B* shows the default network-NAc model. The parameter weights indicate the variation explained in the NAc specifically by each default network subregion (warm/cold colors = positive/negative volume association). The subregions with the strongest volume effect among default network subregions are bilateral orbitofrontal cortex (OFC), left posterior superior temporal sulcus (pSTS), and left ventrolateral prefrontal cortex (vlPFC). Overall, these results suggest there are diverse structural relationships between the NAc and hippocampal subregions, which are substantially similar to the structural relationships of the fornix (Fig. 1). Yet, the relationship of the NAc with default network subregions shows substantially different volume effect sizes for the most prominent cortical regions compared with the fornix. DG, granule cell layer of dentate gyrus (GC-DG-ML; HATA), hippocampal amygdala transition area; ML, molecular layer; Para, parasubiculum; PrS, presubiculum; vmPFC, ventromedial prefrontal cortex.

To complement these hippocampus analyses, the relationship between the NAc and DN subregions was also investigated with a dedicated multiple regression analysis. The subregions with the largest explained variance in NAc volume included right orbitofrontal cortex (OFC), left OFC, left posterior superior temporal sulcus (pSTS), left ventrolateral prefrontal cortex (vlPFC), and left vmPFC (Fig. 2*B*). Conversely, strong negative associations with NAc volume were located to the left and right vmPFC, left and right PCC, as well as right temporal pole. In a Pearson correlation analysis of each DN subregion with the NAc, we also observed that mPFC subregions had generally strong correlations with the NAc and PCC/RSC subregions had generally weak correlations (Supplemental Fig. S4). Overall, in contrast to our HC-centric analyses for the fornix and NAc (cf. above), DN subregions were found to have individually varying relationships with the NAc that noticeably drew a different picture than our results obtained for the fornix-DN relationship.

### Structural Covariation between Hippocampus and Default Network at the Subregion Level

In our core analysis, we explored the principled signatures of structural covariation between the full set of 38 hippocampal subregions and the full set of 91 DN subregions. The concurrent patterns of subregion variation within the hippocampus and within the DN were computed using doubly multivariate pattern-learning analysis. In so doing, we achieved a codecomposition of hippocampal subregion volumes and DN subregion volumes. Each of the top 25 modes of covariation was characterized based on how much of joint variance a particular signature explained, with the most explanatory signature (*mode 1*) achieving a canonical correlation of rho = 0.51 (measured as Pearson’s correlation coefficient) (Supplemental Table S1). The second most explanatory signature (*mode 2*) achieved a canonical correlation of rho = 0.42, the third rho = 0.39, the fourth rho = 0.31, the fifth rho = 0.27, and the sixth rho = 0.23, through to the twenty-fifth signature, which had rho = 0.06 (see Supplemental Table S1 for full list). This analysis thus established the scaffold for all subsequent analyses that delineates how multiple complementary hippocampal patterns covary hand in hand with DN patterns.

### Differences in the Hippocampus-Default Network Covariation in Loneliness

On the basis of the identified population signatures of HC-DN covariation, we investigated the neurobiological manifestations of loneliness in our UKB sample. This was accomplished by examining robust subregion-level divergences in how hippocampal patterns are coexpressed with DN patterns between groups of lonely versus nonlonely participants. To this end, we first analyzed loneliness by a rigorous group difference analysis between the structural patterns of covariation in the lonely and nonlonely groups. This revealed the precise subregions contributing to the structural HC-DN covariation that systematically diverged between the two groups, for each mode of the CCA.

We uncovered a multitude of modes with systematic group differences in either specific HC and/or DN subregions. We also found modes with no significant structural divergences in any hippocampal or DN subregion. From here on, a subregion that was observed to have a robustly different weighting within a mode’s canonical vector, between the lonely and nonlonely groups, is termed a “hit” (i.e., an observed structural divergence in lonely individuals). Across all 25 examined modes, the group contrasts amounted to 28 total hits for HC subregions and 40 total hits for DN regions. Most of these subregion hits occurred in earlier modes. For example, in the first mode we found 26 DN hits (60% of the DN total), and in the first three modes we found 22 HC subregion hits (78.6% of the HC total). We also noted specific subregions with repeated hits across modes: The largest number of hits was in molecular layer body (4 hits), molecular layer head (3), CA1 head (3), CA1 body (3), and presubiculum head (3). In the DN, the greatest number of hits was observed in midline subregions (77.5% of the DN total) such as the RSC and mPFC. We observed few hits in lateral temporal or parietal subregions, with a total number of hits in 5 temporal (12.5%), 13 PFC (32.5%), 2 parietal (5%), and 20 PCC/RSC (50%) subregions.

**Figure 3.**
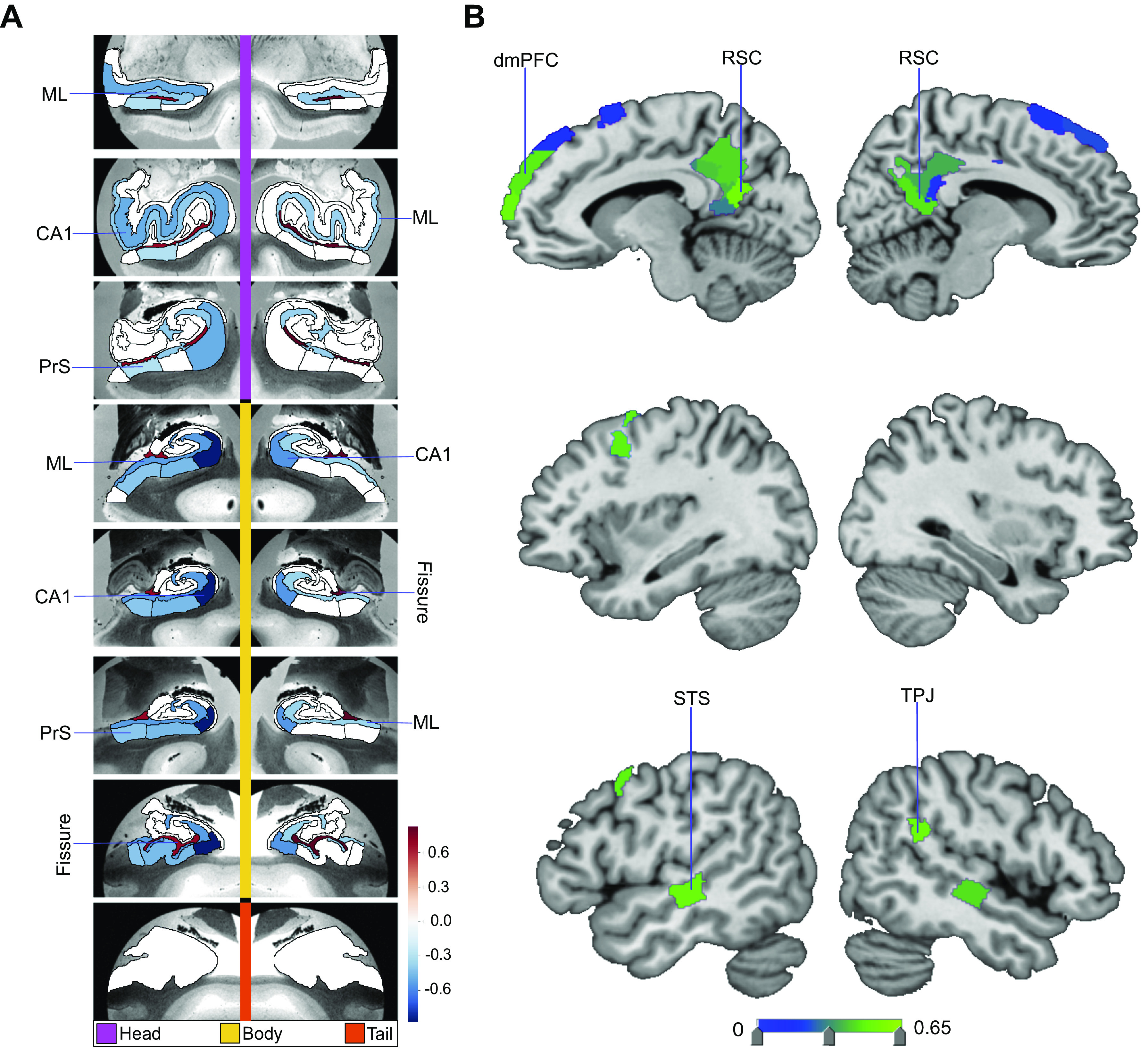
Loneliness is associated with distinct divergences in hippocampus (HC)-default network (DN) covariation. We explored the structural covariation between the 38 subregions of the HC and 91 subregions of the DN, by means of a codecomposition based on a canonical correlation analysis (CCA). We subsequently determined how the ensuing subregion patterns diverged in individuals with loneliness. Shown here are the subregion divergences in *mode 1* of HC-DN covariation. *Mode 1* of the CCA solution achieves the most explanatory covariation, with a canonical correlation of rho = 0.51. *A* shows the HC subregion pattern (left, one canonical vector of *mode 1*) with parameter weights that robustly diverge between lonely and nonlonely groups in *mode 1*; mapped onto 8 consecutive coronal slices of the left and right HC in the anterior (*top*)-to-posterior (*bottom*) direction. *B* shows the DN subregion pattern (right, other canonical vector of *mode 1*) that robustly diverged between the lonely and nonlonely groups. Overall, within the dominant structural covariation pattern between the HC and the DN there are specific subregions whose volumes systematically diverge in lonely individuals. The most pronounced structural divergences are in bilateral CA1 body and hippocampal fissure, as well as subregions bilaterally in the posterior cingulate cortex (PCC), retrosplenial cortex (RSC), and dorsomedial prefrontal cortex (dmPFC). Thus, specific HC and DN anatomical subregions are preferentially linked loneliness. ML, molecular layer; PrS, presubiculum; STS, superior temporal sulcus; Sub, subiculum; TPJ, temporoparietal junction.

**Figure 4.**
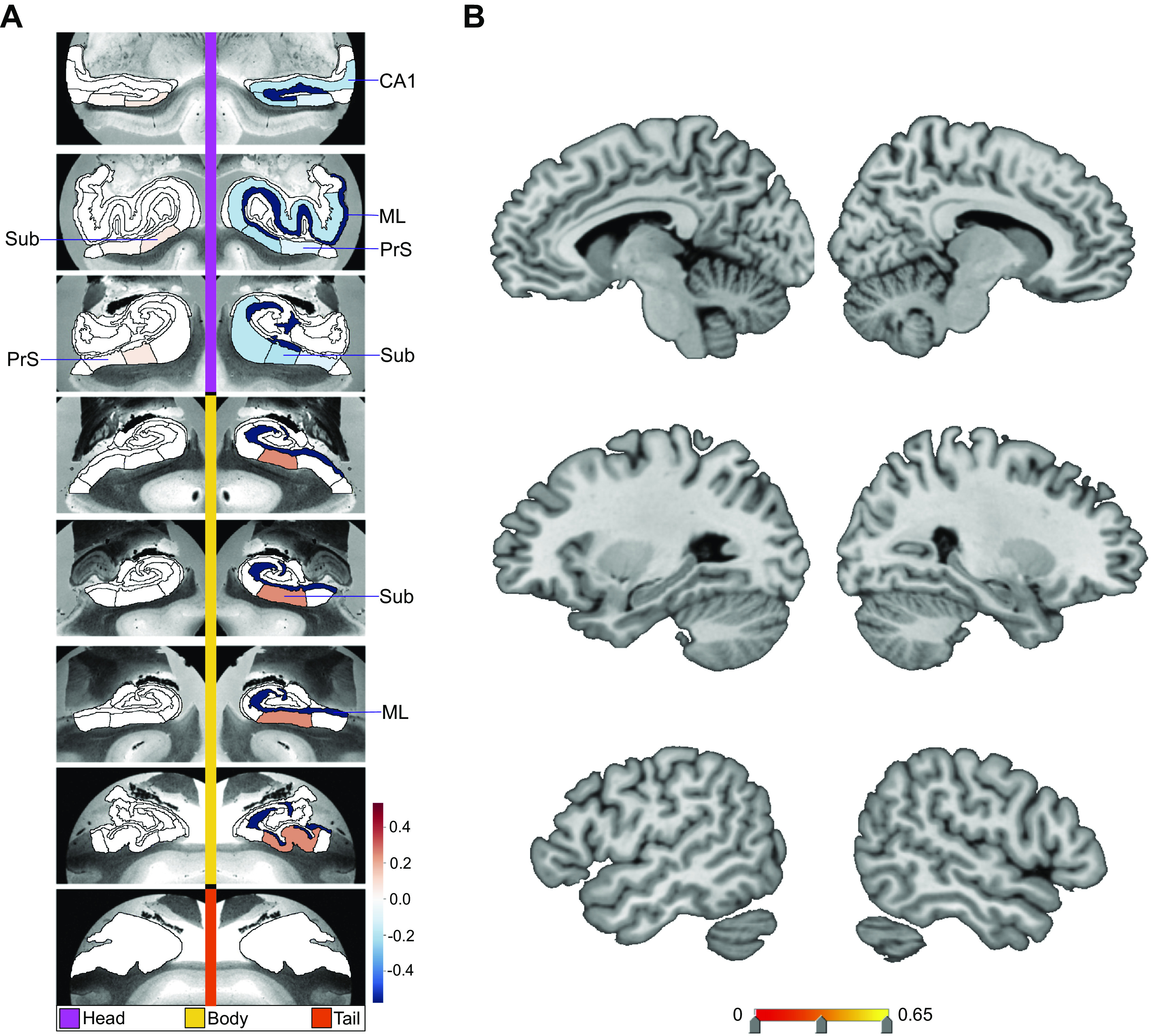
Divergences in CA1, subiculum (Sub), presubiculum (PrS), and molecular layer (ML) subregion volumes are preferentially associated with loneliness. Shown are the subregion divergences in *mode 3* of hippocampus (HC)-default network (DN) covariation. *Mode 3* of the canonical correlation analysis (CCA) solution achieves the third most explanatory hippocampal-DN covariation, with a canonical correlation of rho = 0.39. *A* exhibits the (HC) subregion pattern (left, one canonical vector of *mode 3*) with paramater weights that robustly diverge between lonely and nonlonely groups in *mode 3*. *B* shows the DN subregion pattern (right, other canonical vector of *mode 3*) of the DN (right) that robustly diverge between lonely and nonlonely groups. The most pronounced divergences in the loneliness group are in right ML head and body, right Sub head and body, as well as right CA1 head. Thus, similar to *mode 1*, within the third most explanatory pattern of HC-DN covariation, loneliness is concomitant with hippocampal divergences in CA1, Sub, PrS, and ML.

**Figure 5.**
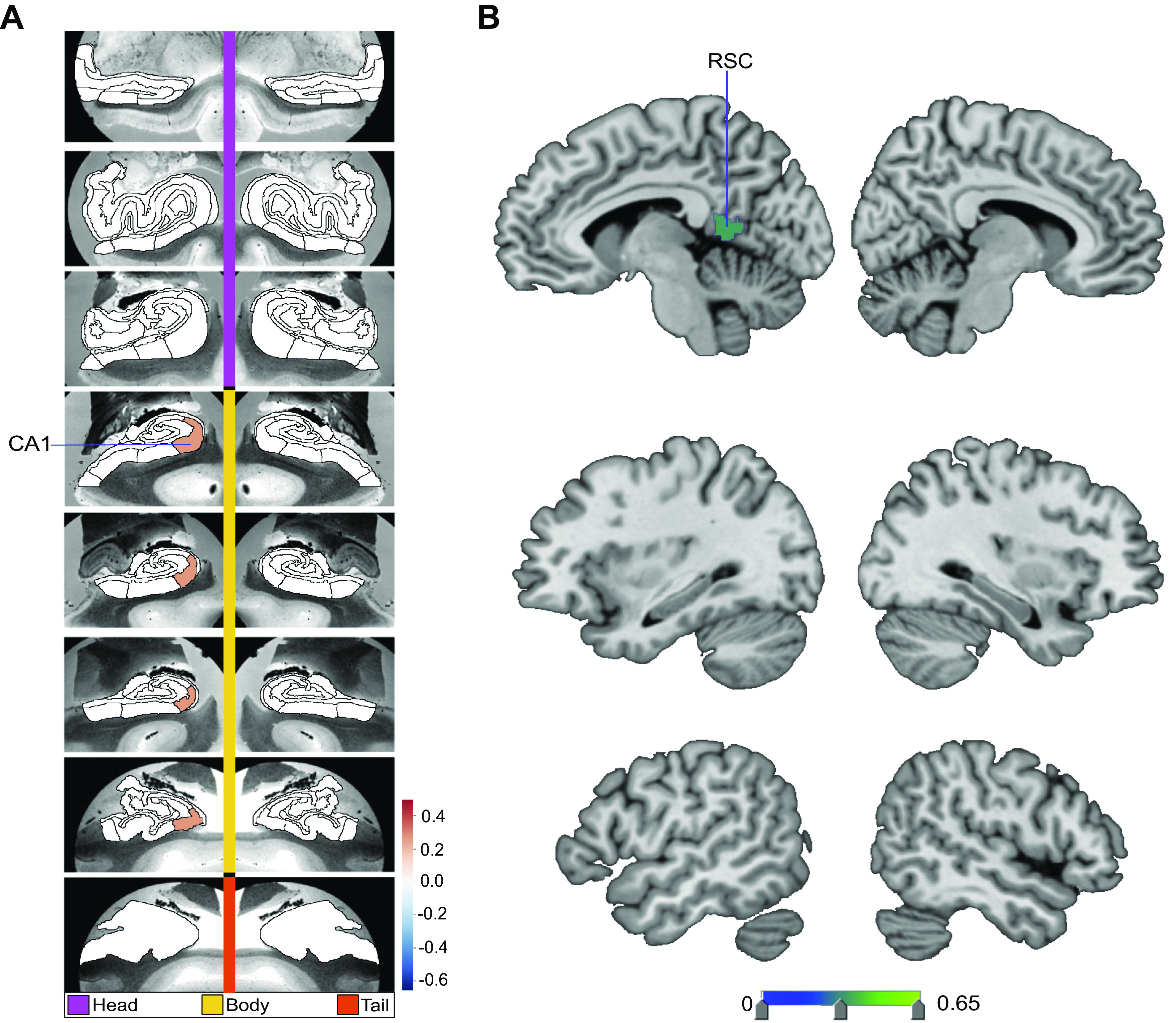
Loneliness is associated with divergences in left hemisphere CA1 and retrosplenial cortex (RSC). Shown are the subregion divergences in *mode 5* of hippocampus (HC)-default network (DN) covariation. *Mode 5* of the canonical correlation analysis (CCA) solution achieves the fifth most explanatory hippocampal-DN covariation, with a canonical correlation of rho = 0.27. *A* exhibits the HC subregion pattern (left, one canonical vector of *mode 5*) with parameter weights that robustly diverge between lonely and nonlonely groups in *mode 5*. *B* shows the DN subregion pattern (right, other canonical vector of *mode 5*) of the DN (right) that robustly diverge between lonely and nonlonely groups. The only subregion divergences associated with loneliness are in left CA1 body and the left RSC. These results accentuate the selectivity of the subregion divergences within a particular mode and highlight CA1 and RSC in loneliness.

**Figure 6.**
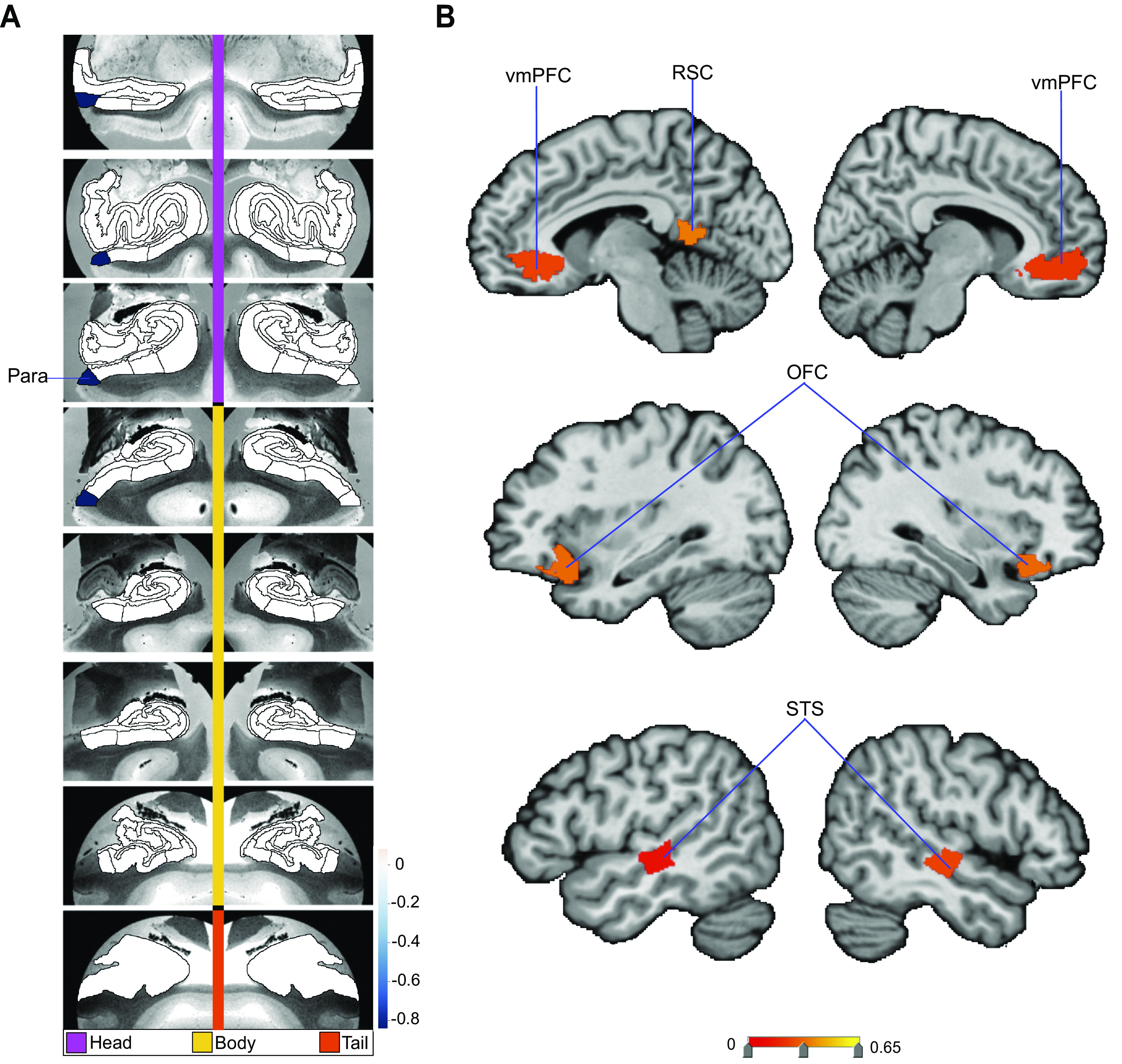
Loneliness is associated with subregion divergences in parasubiculum (Para) and a distributed set of bilateral default network (DN) subregions. Shown here are the subregion divergences in *mode 9* of hippocampus (HC)-DN covariation. *Mode 9* of the CCA solution achieves the ninth most explanatory HC-DN covariation, with a canonical correlation of rho = 0.18. *A* exhibits the HC subregion pattern (left, one canonical vector of *mode 9*) with parameter weights that robustly diverge between lonely and nonlonely groups in *mode 9*. *B* shows the DN subregion pattern (right, other canonical vector of *mode 9*) of the DN (right) that robustly diverged between lonely and nonlonely groups. Overall, there are divergences for lonely individuals in left Para, as well as bilateral ventromedial prefrontal cortex (vmPFC), left retrosplenial cortex (RSC), bilateral orbitofrontal cortex (OFC), and bilateral superior temporal sulcus (STS). These results emphasize the bilateral tendency of the DN divergences observed for loneliness. The results also highlight a coherent set of reward-related subregions, as most of the subregions identified are also found to have a strong volumetric relationship with the nucleus accumbens (Fig. 2).

The constellation of structural divergences between lonely and nonlonely groups in *mode 1* (Fig. 3) provided a rough portrait of the hits that typically emerged across the next 24 modes. In *mode 1* we observed hits in bilateral CA1 head and left CA1 body, the adjacent left presubiculum head and body, as well as the bilateral subregions internal to the hippocampus (ML and fissure). Concurrently, the DN hits for *mode 1* were clustered in adjacent subregions in the bilateral PCC/RSC region and mPFC (2 temporal, 8 PFC, 1 parietal, 15 PCC). Additionally, the subregions with hits that played an especially strong role in the dominant mode’s patterns were bilateral CA1 body, bilateral fissure, bilateral RSC, and left dmPFC. In contrast, for the second mode of the HC-DN covariation, we observed hits only in the left parasubiculum and left ML head, with no hits in DN regions (Supplemental Fig. S5). For *mode 3*, we observed eight HC hits and no DN hits (Fig. 4); for *mode 4*, one hit in the HC tail and no DN hits for *mode 5*, one HC and one DN hit (Fig. 5); for *mode 6*, no hits; for *mode 7*, no HC hits and one hit in the right temporoparietal junction for *mode 8*, HC hits in three subregions, the left CA1 head, left CA2/3 head, and left granule cell layer of dentate gyrus (GC-ML-DG) head, and no DN hits (Supplemental Fig. S6); and for *mode 9*, one HC hit and seven DN hits (Fig. 6). We observed no hits in *modes 10–13*. In *mode 14* we found no HC hits and three DN hits: left superior temporal sulcus (STS), right anterior cingulate cortex (ACC), and left RSC (Supplemental Fig. S7). In *mode 15*, we found no HC hits and two hits in DN subregions: bilateral PCC (Supplemental Fig. S8). Of note, we observed no hits in any of the modes between *mode 16* and *mode 25*. Thus the dominant modes, which account for more population covariance, are most strongly coupled with loneliness.

These results suggest that specific subregions that play a role in the HC-DN correspondence exhibit systematic diverges in individuals with loneliness. Additionally, our findings specify within which context of structural dependence (mode of covariation) a subregion diverged between the two groups. Overall, across loneliness’s relationships to our discovered population modes of joint volume variation, CA1 head and body, molecular layer head and body, and presubiculum head showed the largest number of hits for hippocampal subregions. On the other hand, there was a preponderance of hits located to midline cortical structures, such as the mPFC and PCC/RSC, for DN subregions. Thus, our population-level findings made apparent that within structural covariation modes, loneliness was mostly concomitant with divergences in DN midline subregions that were grounded in parallel divergences in the CA1 and ML subregions of our hippocampus atlas.

### Genetic Predisposition for Loneliness

**Figure 7.**
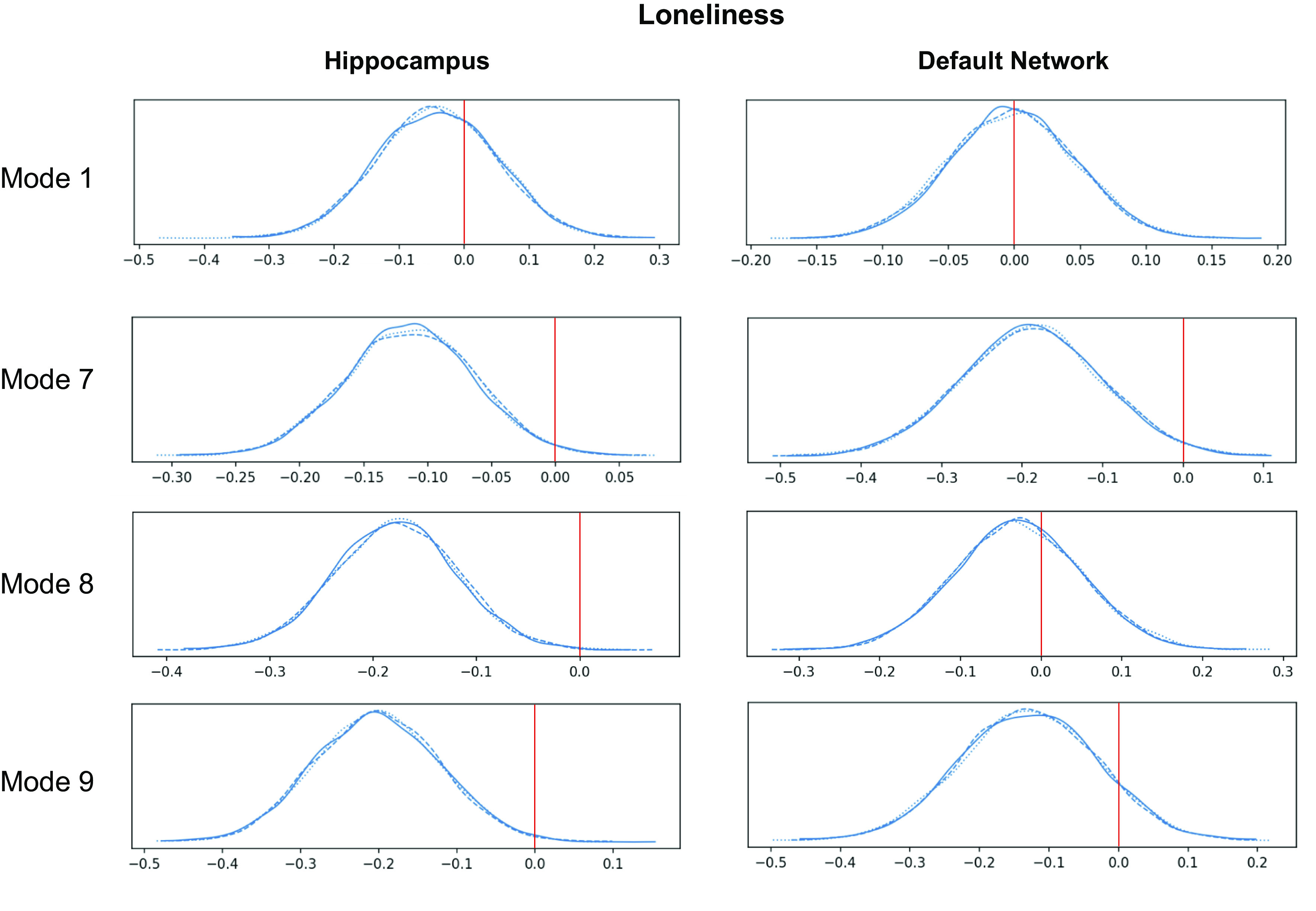
The genetic predisposition for loneliness is associated with the expression of specific hippocampus(HC)-default network (DN) covariation patterns. A polygenic risk score (PRS) analysis was conducted to estimate the subject-specific heritable tendency for loneliness based on genome-wide effects in single-nucleotide polymorphisms. The subject-specific PRS estimates were then regressed against the expressions of each of the modes of structural covariation between the HC and DN. The relevance of the heritability effects was judged based on the posterior parameter distributions inferred by the Bayesian logistic regression model (histograms). The *x*-axis of each plot represents the β coefficient of the model parameter, and the *y*-axis of each plot represents the plausibility of each coefficient value. A value of 0 indicates no association between PRS and interindividual mode expression. Overall, loneliness predisposition was related to the expression of *modes 7* and *9* on the HC side and to *mode 7* on the DN network side. These results demonstrate that specific mode expressions are robustly linked to the predisposition of becoming lonely. The three posterior distribution histograms show convergence across three different Markov chain Monte Carlo runs. Overall, this pinpoints the specific neurobiological signatures in the HC and DN that relate to the heritable components of loneliness.

We finally sought to interrogate whether the uncovered HC-DN covariation expressions featured systemic relationships with the participants’ liability for loneliness (cf. materials and methods). For this purpose, we first computed polygenic risk score predictions of loneliness risk for our UK Biobank participants. We observed that there was a statistically relevant relationship between loneliness PRS score and participant expressions (i.e., canonical variates) of *modes 7*, *8*, *9*, and *22* in the hippocampus and the participant expressions of *mode 7* in the DN (Fig. 7). On the whole, there was genetic predisposition linked to the expressions of later modes compared with the earlier modes achieving greater explained variance. This analysis suggested that the expression of the identified HC-DN signatures tracks the purely heritable components of loneliness due to single-nucleotide polymorphism. As a matter of course, the signatures not identified likely have contributions that are not due to genetic factors.

## DISCUSSION

We have tailored an analytical framework to examine how the structural substrates of the HC-DN correspondence systematically deviate in loneliness. We work toward this goal by directly estimating principled covariation signatures that delineate how hippocampus subregion volumes are coexpressed with DN subregion volumes in ∼40,000 participants from the UK Biobank cohort. In so doing, our study aimed to refine the understanding of the interrelationship of the DN with loneliness. By anchoring our analysis in HC-DN covariation, we aimed to facilitate the interpretation of potentially human-specific DN brain regions based on their hippocampal subregion affiliates. Indeed, the evolutionarily conserved hippocampus has been extensively studied in animal species at a single-cell resolution. This approach can detect patterns in the structural brain scans that are not discoverable by any analysis of the HC or the DN alone.

The DN has recently been found to be the major brain network that is most closely associated with loneliness (29). However, we found a high degree of heterogeneity in the relationship of individual DN subregions with loneliness in the context of population modes of HC-DN volume covariation. In particular, we found that especially midline structures of the DN tended to diverge in lonely individuals. Analogously, the hippocampal subregions with the most divergences across modes were CA1 and molecular layer. The allocortical-neocortical divergences implicating midline DN as well as the CA1 and molecular layer of the HC stood out, especially in *modes 1*, *3*, and *5* of our analysis. The concomitant structural divergences of HC and DN subregions in lonely individuals may in part be explained by the varieties of internally directed cognition that have been consistently associated with each neural system—such as episodic memory processing, sense of self, and prospective cognition (21, 75–77). Especially midline structures of the DN such as the vmPFC (78), posteromedial cortex (79, 80), and the RSC (75) have been found to serve these types of neural processes. In line with this notion, the fornix—known to be a direct mediator between the HC and midline DN subregions—has been found to be the white matter structure that is most strongly linked to loneliness profiles (29). This observation is of relevance to our present considerations, as direct neuron-to-neuron connections between CA1 and subiculum of the hippocampus with mPFC, OFC, and RSC subregions of the DN have been demonstrated through invasive axonal tracing studies in monkeys (28, 81–84). Human studies have also found that the functional connectivity strength between the hippocampus and mPFC correlates with the imagination of mental scenes (78, 85, 86) and precommissural fornix integrity correlates with episodic richness (87, 88). Indeed, these earlier findings may explain various aspects of the psychological alterations that are characteristic for lonely individuals.

For example, lonely individuals have been described to more frequently reiterate social events from the past, imagine hypothetical or future encounters with others, and reminisce on nostalgic memories (89–93). The joint divergences of CA1 and midline DN subregions thus suggest an intimate link between these spatially distributed subregions, a link that *1*) is supported by the fornix, *2*) implicates internally directed cognition, and *3*) is systematically altered in lonely individuals. The notion of CA1, molecular layer, OFC, and mPFC subregions having preferential structural relationships with the fornix receives additional support from our multiple regression and pairwise Pearson’s correlation analyses. Indeed, each of these subregions showed especially strong relationships with the fornix in both analyses. In accord with the growing knowledge of the fornix, it is possible that these concordant findings reflect a difference in the neurocognitive processes necessary for episodic memory retrieval (88, 94). For example, the CA1 and subiculum, which are the primary origin of precommissural fornix projection fibers to the PFC and NAc (28, 30, 95), have been found to be crucial for proto-episodic memory in rodents (27, 96) and detailed episodic autobiographical memory in humans (97, 98). Hence, the known cognitive upregulation in memory retrieval and social perception biases of lonely individuals may be rooted in how the fornix pathway assists HC-DN communication.

The cognitive biases of the loneliness trait further include perceiving one’s social world as a more threatening place (4, 99), such as remembering more negative information from past social encounters and expecting more negative social interactions (5). On the basis of these earlier psychological insights, we expected that the subregions with multiple divergences in lonely individuals would show a strong relationship to the NAc, a key node of the brain’s reward circuitry that has previously been associated with loneliness (31). In support of this idea, our multiple regression analysis revealed that CA1, molecular layer, vmPFC, and OFC each showed strong relationships with the NAc. The seemingly separate psychological and behavioral characteristics of loneliness may thus be a product of the remodeling of integrated neural systems. This notion receives further support from the similarity in the subregion associations revealed through our separate multiple regression models of the hippocampus. In these, we observed a strong overlap between the hippocampal subregions having strong relationships with either the NAc or fornix. Indeed, the NAc receives the bulk of its hippocampal inputs through the precommissural fornix pathway (83, 100). Hence, our results substantiate previous findings on cross-associations between the CA1 of the hippocampus, fornix, mPFC, and NAc.

In addition to the collective divergences in hippocampal and mPFC subregions in lonely individuals, our analysis pinpointed parallel findings in other DN subregions. In particular, the majority of DN subregion divergences we found were in the PCC, and especially RSC. In general, these subregions also showed strong structural relationships with the fornix in our regression analyses. In line with our results from brain imaging modeling, direct axon tracing studies in rodents have documented CA1 and subiculum as the main hippocampal projection sites to the RSC (101, 102). Monkey experiments have additionally reported that direct HC-RSC projections are chiefly mediated through the subiculum, a close interaction partner of hippocampal CA1 (84, 102–104). In conjunction with the CA1 structural divergences we observed in lonely individuals, we also observed four total subiculum divergences across all examined signatures. Hence, our population neuroscience evidence for interdependence between CA1 and subiculum in the allocortex and RSC subregions in the neocortex directly confirms established knowledge from invasive animal experiments (105). Thus, an attractive interpretation of these findings of structural divergences in RSC subregions for lonely individuals (in *modes 1*, *5*, *9*, *14*, and *15*) invokes a difference in the capacity to visualize internally centered thoughts. Indeed, among the limited knowledge regarding the function of the human RSC, this part of the posterior midline is believed to assist in spatial navigation, episodic memory, visualizing details of mental scene construction, and perhaps mentalizing capability (21, 26, 106–108).

Moreover, RSC-mediated mental scene construction could be one aspect of a more general role the DN has in spontaneous cognition, such as task-free “mind-wandering” (109, 110). In fact, the hippocampus has been found to support stimulus-independent neural activity (111), which would be a perfect partner for these DN functions, via so-called sharp-wave ripples (SWRs) (112, 113). These phenomena are manifestations of spontaneous neural activity synchronizations in the hippocampus circuit that occur independent of any environmental cues or externally structured tasks (114, 115). SWRs have been carefully studied through electrophysiological experiments in rodents and other animals, as opposed to most of the neural activity specific to regions of the human DN (27, 116). Of relevance for our present considerations, CA1 pyramidal neurons are purported to underlie the expansive “ripples” of SWR activity. The preceding “sharp wave,” on the other hand, is most pronounced in the apical dendrites of CA1 neurons (27). Along this line, the hippocampus atlas used for our study collapsed the apical dendrites (both the stratum radiatum and stratum lacunosum moleculare) of subiculum and CA neurons into one composite subregion: the molecular layer (40). Thus, the molecular layer and CA1 of our set of human hippocampus subregions offered a way to analyze systematic alterations of the neural substrates shown to be highly visible locations of SWR activity in the hippocampus of rodents and monkeys.

Indeed, the CA1 and molecular layer had the greatest number of total hits in lonely individuals. More specifically, there were seven total hits in the molecular layer and six total hits in CA1 across all examined modes. Since our analyses revealed co-occurring divergences in midline DN subregions, SWR activity suggests itself as a compelling interpretation that can accommodate these collective results. The sensory-distal processing regimes realized by the DN may be perfectly attuned to integrating spontaneous SWR activity. For example, in rodents, CA1 cell activity and coupled SWR have been shown to assist in prospective cognitive processes (117), such as simulating spatial trajectories that were never experienced before (118–120). A functional interaction between hippocampal SWR activity and association cortex regions, such as the RSC, has even been demonstrated in animal species (105, 121). For example, in rats it has been shown that auditory cortex activity predicts subsequent SWR activity (122). Conversely, hippocampal activity also predicted subsequent activity in the auditory cortex. On the basis of these results, the authors proposed that immediately before and after SWR occur there is rapid information flow in a cortical-hippocampal-cortical loop (122). Similarly, studies in primates (123, 124) have also identified the RSC and PCC to be the regions of the neocortical mantle that are most closely coupled with hippocampal SWR (124). Our findings of selective structural divergences in both the CA1 and molecular layer as well as RSC subregions across modes is thus very suggestive of a difference in the allocortical-neocortical information processing pathways that involve SWR activity.

Facilitation of mental scene construction is discussed as one of the main functional contributions of the hippocampus (21, 109). It thus appears plausible that a systematic alteration of SWR activity may affect the vividness of an individual’s episodic memories and imaginings (125). In fact, SWR activity has been proposed to underlie trajectory sequence replay in rodents as well as “offline” states of human cognition, such as daydreaming (32). Thus, the increased frequency and intensity of nostalgic reminiscence that has been associated with loneliness may potentially also be associated with altered SWR activity (91). A strong relationship between SWR and loneliness is additionally suggested by their shared association with reward processing. Indeed, loneliness alters the subjectively perceived valence of social and nonsocial reward in humans (7), while SWR activity has been demonstrated to be responsive to reward-contingent processing in animals (126–128). Similarly, the fundamental place cell representations of the CA1 pyramidal cells, which underlie SWR activity (cf. above), were reported to intimately relate to reward processing (129, 130). Certain populations of CA1 cells have even been found to act as a dedicated channel for reward-related processing across environments (131). These findings and conclusions from previous animal experiments therefore appear to dovetail with our analyses in people highlighting the NAc with CA1 and molecular layer. We hence wonder whether the attempt to fill the perceived social void through mental imagery in lonely individuals coincides with interlocked structural alterations in the allocortical-neocortical covariations.

However, the relationship between the structure of the HC and DN and the tendency to feel socially disconnected from others may only be partially determined by one’s environment and life experience. Indeed, our genome-wide analyses showed that participant-specific expressions of HC-DN covariation have distinct links to the heritable components of loneliness. This is in accord with previous research showing that loneliness has consistent, yet subtle genetic underpinnings (132). For example, twin studies have identified genetic contributions to individual differences in feelings of loneliness that are as high as ∼48% (133). Recent genome-wide association studies with large sample sizes [e.g., *n* = 452,302 (134)] have also reported the contribution of common genetic variants to loneliness to range from 4% to 27% (134–136). These earlier genome-wide analyses have additionally pointed out a small number of specific gene loci that are significantly associated with loneliness and regular participation in social groups (134).

An underlying genetic contribution to the experience of loneliness is further supported by recent genome-wide correlation analyses. For example, one study has demonstrated that our UK Biobank loneliness trait significantly shared underlying genetic factors with each of 264 different demographic, lifestyle, and disease phenotypes (29). Another study has demonstrated a strong genetic correlation between loneliness and neuroticism (136). Taken together, these recent population neuroscience studies indicate that the genetic determinants underlying loneliness are probably quite polygenic and involve complex gene-environment interactions. In our study, these previous insights were extended by relating PRS for loneliness with the interindividual expression of multiple spatially overlapping signatures of HC-DN covariation. Importantly, we have identified a select subset of HC-DN covariation modes that had a significant relationship with genetic predisposition for loneliness.

In particular, we found a significant heritability effect for the expression of the loneliness-specific signature in *modes 8* and *9*, both of which also showed multiple subregion divergences in lonely individuals. We also noted a particular concordance between our PRS analysis of loneliness with our uncovered subregion-specific divergences in *mode 9*. Intriguingly, in our multiple regression analyses of the NAc, each subregion that showed divergence in *mode 9* for loneliness (i.e., OFC, vmPFC, pSTS, and left parasubiculum) was found to have a strong relationship with the NAc. These convergent results may suggest that a specific aspect of the genetic predisposition for loneliness consists in innate tendencies involving reward-related processing. This view would be in line with past findings associating loneliness with social reward valence (7), substance abuse or dependence (12, 137), executive function (138, 139), and impulsive behaviors (140). Recently, it has even been reported that social isolation is linked to altered neural activity responses in midbrain regions and induces social craving in a way that may be similar to how fasting causes hunger (141). These reports further motivate a suspicion that the unmet desire for social connection in people is related to the same reward mechanisms in nonsocial reward. More specifically, the β-endorphin system, which is involved in pain management and reward valence, has also been shown to be integral to social bonding through touch in multiple mammalian species (142–144).

However, our findings on how structural HC-DN covariation relates to the genetic predisposition for social isolation may be better framed in terms of susceptibility to the environment, such as the previously proposed notion that the driving forces behind genetic heritability are not direct but rather come into effect through an altered sensitivity to environmental conditions (145). For example, whereas oxytocin and social support have been shown to interact in the stress response (146), a single-nucleotide polymorphism in the oxytocin receptor gene (*OXTR*) has been found to differentially affect stress response depending on adequate social interactions with others (147). Indeed, the heightened levels of stress in humans with social isolation has been argued to be fundamentally different from a simple general and diffuse stress response (6, 148).

In fact, animal studies have afforded detailed accounts of the effects of social isolation stress on the hippocampus (149), focusing on the apical dendrites of CA neurons (150). When considered in conjunction with this animal literature, our analysis could therefore extend our incomplete understanding of the brain basis of social isolation-related stress by identifying the precise allocortical and neocortical anatomical subregions at play. A possible manifestation of the effects of social isolation-related stress in our study is the pronounced total number of divergences in the molecular layer. In particular, we found seven total hits in the molecular layer across all examined modes for lonely individuals. As mentioned above, this molecular layer subregion consisted of a combination of the apical dendrites of subiculum and CA neurons, with CA1 composing the largest portion (40). In light of this, the observation of preferential hits in the molecular layer of lonely individuals broadly aligns with past studies on chronic stress in rodents, tree shrews, and nonhuman primates. Indeed, experimental studies in these species have reported detailed cellular and physiological consequences of induced stress on the hippocampus and other related brain regions.

In the hippocampus specifically, it has been shown that chronic stress results in the selective atrophy of the apical dendrites of CA3 cells but not CA3 basal dendrites (151–155). In contrast, elevated chronic stress in the face of multiple stressors results in additional atrophy of the apical dendrites of CA1 cells yet not CA1 basal dendrites (156, 157). On the basis of these findings, researchers have proposed that CA3 apical dendrites are the most susceptible hippocampal structure to chronic stress. Yet, stress affects CA1 apical dendrites only with a more severe experimental stressor than that needed to affect CA3 apical dendrites (150). Our findings of preferential hits in the molecular layer of lonely individuals may thus be a marker of elevated stress and relate to the physiological characterization of loneliness as a trait coinciding with elevated stress and immune response levels (5, 8, 158–161).

Building upon this, our concerted analysis framework for HC and DN subregion variation thus allowed us to relate the potential effects of chronic stress on neocortical partners in the DN. For example, in *mode 1* we observed four hits in the molecular layer with coincident hits in midline DN subregions such as the dmPFC, PCC, and RSC. The collection of parallel findings of divergences between the stress-susceptible molecular layer in the allocortex and specific midline cortical subregions might therefore reflect the effects of stress on the higher associative cortex.

Indeed, previous studies have shown that the effects of chronic stress on the hippocampus percolate into interaction partners with known axonal connections, such as target areas in the mPFC (28, 81, 82, 162, 163). For example, in one study where rats were submitted to 4 wk of chronic stress, they were found to have a deficit in hippocampal-PFC synaptic strength compared with control animals (162). It was also found that the stressed rats had lower PFC volume and poorer performance on working memory and behavioral flexibility tasks—activities that are thought to depend on mPFC activity (162). Another more recent study has demonstrated that among squirrel monkeys with bouts of prepubertal social isolation, there was an association between functional hyperconnectivity in PFC-subcortical circuits (e.g., dmPFC, vmPFC, OFC; amygdala, ventral striatum, HC) with reduced anxiety-like behavior in later adulthood. In line with these previous experiments, our analytical strategy in the UK Biobank population allowed us to identify concomitant stress-related divergences in the molecular layer of the hippocampus and divergences in the mPFC, PCC, and RSC of the human DN.

It is well known that chronic stress causes measurable neural consequences for the hippocampus in different animal species (164–166). Yet, the impact of social isolation and chronic stress on the hippocampus has repeatedly been argued to be reversible in a matter of a few weeks with social rehabilitation or reintroduction to an enriched environment (33, 34, 167, 168). In a similar vein, in the higher association cortex the effects of chronic stress on PFC dendritic morphology in rats and functional connectivity in humans have been shown to be reversible after stressful experiences (169–172). In light of this past literature, there is likely to be elasticity to the effects of social isolation and perhaps the stress-related volumetric divergences that we uncovered in the hippocampus and its neocortical interaction partners. Indeed, social rehabilitation by returning to the usual social environment may remold the examined allocortical-neocortical brain circuits.

## SUPPLEMENTAL DATA

Supplemental Figs. S1–S9 and Supplemental Table S1: https://doi.org/10.6084/m9.figshare.15060684.

## GRANTS

This project has been made possible by the Brain Canada Foundation, through the Canada Brain Research Fund, as well as by NIH Grant R01AG068563A and the Canadian Institutes of Health Research. D.B. was also supported by the Healthy Brains Healthy Lives initiative (Canada First Research Excellence fund) and by the CIFAR Artificial Intelligence Chairs program (Canada Institute for Advanced Research) as well as a Research Award and Teaching Award by Google.

## DISCLOSURES

No conflicts of interest, financial or otherwise, are declared by the authors.

## AUTHOR CONTRIBUTIONS

C.Z., R.N.S., and D.B. conceived and designed research; D.B. performed experiments; C.Z. and D.B. analyzed data; C.Z. and D.B. interpreted results of experiments; C.Z. and D.B. prepared figures; C.Z. and D.B. drafted manuscript; C.Z., R.N.S., and D.B. edited and revised manuscript; C.Z., R.N.S., and D.B. approved final version of manuscript.

## References

[1] Holt-Lunstad J, Smith TB, Baker M, Harris T, Stephenson D. Loneliness and social isolation as risk factors for mortality: a meta-analytic review. Perspect Psychol Sci 10: 227–237, 2015. doi:10.1177/1745691614568352. 25910392

[2] Bzdok D, Dunbar RIM. The neurobiology of social distance. Trends Cogn Sci 24: 717–733, 2020. doi:10.1016/j.tics.2020.05.016. 32561254 PMC7266757

[3] Beutel ME, Klein EM, Brähler E, Reiner I, Jünger C, Michal M, Wiltink J, Wild PS, Münzel T, Lackner KJ, Tibubos AN. Loneliness in the general population: prevalence, determinants and relations to mental health. BMC Psychiatry 17: 97, 2017. doi:10.1186/s12888-017-1262-x. 28320380 PMC5359916

[4] Cacioppo JT, Hawkley LC. Perceived social isolation and cognition. Trends Cogn Sci 13: 447–454, 2009. doi:10.1016/j.tics.2009.06.005. 19726219 PMC2752489

[5] Hawkley LC, Cacioppo JT. Loneliness matters: a theoretical and empirical review of consequences and mechanisms. Ann Behav Med 40: 218–227, 2010. doi:10.1007/s12160-010-9210-8. 20652462 PMC3874845

[6] Cacioppo JT, Cacioppo S. Loneliness in the modern age: an evolutionary theory of loneliness (ETL). Adv Exp Soc Psychol 58: 127–197, 2018. doi:10.1016/bs.aesp.2018.03.003.

[7] Cacioppo JT, Norris CJ, Decety J, Monteleone G, Nusbaum H. In the eye of the beholder: individual differences in perceived social isolation predict regional brain activation to social stimuli. J Cogn Neurosci 21: 83–92, 2009. doi:10.1162/jocn.2009.21007. 18476760 PMC2810252

[8] Cacioppo JT, Ernst JM, Burleson MH, McClintock MK, Malarkey WB, Hawkley LC, Kowalewski RB, Paulsen A, Hobson JA, Hugdahl K, Spiegel D, Berntson GG. Lonely traits and concomitant physiological processes: the MacArthur social neuroscience studies. Int J Psychophysiol 35: 143–154, 2000. doi:10.1016/s0167-8760(99)00049-5. 10677643

[9] Cole SW, Capitanio JP, Chun K, Arevalo JM, Ma J, Cacioppo JT. Myeloid differentiation architecture of leukocyte transcriptome dynamics in perceived social isolation. Proc Natl Acad Sci USA 112: 15142–15147, 2015. doi:10.1073/pnas.1514249112. 26598672 PMC4679065

[10] Cacioppo JT, Cacioppo S, Capitanio JP, Cole SW. The neuroendocrinology of social isolation. Annu Rev Psychol 66: 733–767, 2015. doi:10.1146/annurev-psych-010814-015240. 25148851 PMC5130104

[11] Cacioppo JT, Cacioppo S. The growing problem of loneliness. Lancet 391: 426, 2018. doi:10.1016/S0140-6736(18)30142-9. 29407030 PMC6530780

[12] Åkerlind I, Hörnquist JO. Loneliness and alcohol abuse: a review of evidences of an interplay. Soc Sci Med 34: 405–414, 1992. doi:10.1016/0277-9536(92)90300-f. 1566121

[13] Caspi A, Harrington H, Moffitt TE, Milne BJ, Poulton R. Socially Isolated children 20 years later: risk of cardiovascular disease. Arch Pediatr Adolesc Med 160: 805–811, 2006. doi:10.1001/archpedi.160.8.805. 16894079

[14] Hawkley LC, Burleson MH, Berntson GG, Cacioppo JT. Loneliness in everyday life: cardiovascular activity, psychosocial context, and health behaviors. J Pers Soc Psychol 85: 105–120, 2003. doi:10.1037/0022-3514.85.1.105. 12872887

[15] Lara E, Martín-María N, De la Torre-Luque A, Koyanagi A, Vancampfort D, Izquierdo A, Miret M. Does loneliness contribute to mild cognitive impairment and dementia? A systematic review and meta-analysis of longitudinal studies. Ageing Res Rev 52: 7–16, 2019. doi:10.1016/j.arr.2019.03.002. 30914351

[16] Wilson RS, Krueger KR, Arnold SE, Schneider JA, Kelly JF, Barnes LL, Tang Y, Bennett DA. Loneliness and risk of Alzheimer disease. Arch Gen Psychiatry 64: 234–240, 2007. doi:10.1001/archpsyc.64.2.234. 17283291

[17] Holwerda TJ, Deeg DJ, Beekman AT, van Tilburg TG, Stek ML, Jonker C, Schoevers RA. Feelings of loneliness, but not social isolation, predict dementia onset: results from the Amsterdam Study of the Elderly (AMSTEL). J Neurol Neurosurg Psychiatry 85: 135–142, 2014. doi:10.1136/jnnp-2012-302755. 23232034

[18] Braak H, Braak E. Evolution of the neuropathology of Alzheimer’s disease. Acta Neurol Scand Suppl 94: 3–12, 1996. doi:10.1111/j.1600-0404.1996.tb05866.x. 8740983

[19] Buckner RL, Andrews-Hanna JR, Schacter DL. The brain’s default network: anatomy, function, and relevance to disease. Ann NY Acad Sci 1124: 1–38, 2008. 18400922 10.1196/annals.1440.011

[20] Sperling RA, Dickerson BC, Pihlajamaki M, Vannini P, LaViolette PS, Vitolo OV, Hedden T, Becker JA, Rentz DM, Selkoe DJ, Johnson KA. Functional alterations in memory networks in early Alzheimer’s disease. Neuromolecular Med 12: 27–43, 2010. doi:10.1007/s12017-009-8109-7. 20069392 PMC3036844

[21] Hassabis D, Maguire EA. The construction system of the brain. Philos Trans R Soc Lond B Biol Sci 364: 1263–1271, 2009. doi:10.1098/rstb.2008.0296. 19528007 PMC2666702

[22] Hassabis D, Kumaran D, Vann SD, Maguire EA. Patients with hippocampal amnesia cannot imagine new experiences. Proc Natl Acad Sci USA 104: 1726–1731, 2007. doi:10.1073/pnas.0610561104. 17229836 PMC1773058

[23] Burgess N, Maguire EA, O’Keefe J. The human hippocampus and spatial and episodic memory. Neuron 35: 625–641, 2002. doi:10.1016/S0896-6273(02)00830-9. 12194864

[24] Zawadzki MJ, Graham JE, Gerin W. Rumination and anxiety mediate the effect of loneliness on depressed mood and sleep quality in college students. Health Psychol 32: 212–222, 2013. doi:10.1037/a0029007. 22823068

[25] Campbell WK, Krusemark EA, Dyckman KA, Brunell AB, McDowell JE, Twenge JM, Clementz BA. A magnetoencephalography investigation of neural correlates for social exclusion and self-control. Soc Neurosci 1: 124–134, 2006. doi:10.1080/17470910601035160. 18633781

[26] Andrews-Hanna JR, Saxe R, Yarkoni T. Contributions of episodic retrieval and mentalizing to autobiographical thought: evidence from functional neuroimaging, resting-state connectivity, and fMRI meta-analyses. Neuroimage 91: 324–335, 2014. doi:10.1016/j.neuroimage.2014.01.032. 24486981 PMC4001766

[27] Buzsáki G. Hippocampal sharp wave‐ripple: a cognitive biomarker for episodic memory and planning. Hippocampus 25: 1073–1188, 2015. doi:10.1002/hipo.22488. 26135716 PMC4648295

[28] Aggleton JP, Wright NF, Rosene DL, Saunders RC. Complementary patterns of direct amygdala and hippocampal projections to the macaque prefrontal cortex. Cereb Cortex 25: 4351–4373, 2015. doi:10.1093/cercor/bhv019. 25715284 PMC4612443

[29] Spreng RN, Dimas E, Mwilambwe-Tshilobo L, Dagher A, Koellinger P, Nave G, Ong A, Kernbach JM, Wiecki TV, Ge T, Li Y, Holmes AJ, Yeo BT, Turner GR, Dunbar RI, Bzdok D. The default network of the human brain is associated with perceived social isolation. Nat Commun 11: 6393, 2020. doi:10.1038/s41467-020-20039-w. 33319780 PMC7738683

[30] Friedman DP, Aggleton JP, Saunders RC. Comparison of hippocampal, amygdala, and perirhinal projections to the nucleus accumbens: combined anterograde and retrograde tracing study in the macaque brain. J Comp Neurol 450: 345–365, 2002. doi:10.1002/cne.10336. 12209848

[31] Kiesow H, Uddin L, Bernhardt B, Kable J, Bzdok D. Dissecting the midlife crisis: disentangling social, personality and demographic determinants in social brain anatomy (Preprint). *bioRxiv* 423702, 2020. doi:10.1101/2020.12.20.423702. PMC8211729 34140617

[32] Buzsáki G. The Brain from Inside Out. New York: Oxford University Press, 2019.

[33] Biggio F, Mostallino M, Talani G, Locci V, Mostallino R, Calandra G, Sanna E, Biggio G. Social enrichment reverses the isolation-induced deficits of neuronal plasticity in the hippocampus of male rats. Neuropharmacology 151: 45–54, 2019. doi:10.1016/j.neuropharm.2019.03.030. 30935859

[34] Ibi D, Takuma K, Koike H, Mizoguchi H, Tsuritani K, Kuwahara Y, Kamei H, Nagai T, Yoneda Y, Nabeshima T, Yamada K. Social isolation rearing‐induced impairment of the hippocampal neurogenesis is associated with deficits in spatial memory and emotion‐related behaviors in juvenile mice. J Neurochem 105: 921–932, 2008. doi:10.1111/j.1471-4159.2007.05207.x. 18182044

[35] Silva-Gómez AB, Rojas D, Juárez I, Flores G. Decreased dendritic spine density on prefrontal cortical and hippocampal pyramidal neurons in postweaning social isolation rats. Brain Res 983: 128–136, 2003. doi:10.1016/s0006-8993(03)03042-7. 12914973

[36] Kogan JH, Frankland PW, Silva AJ. Long‐term memory underlying hippocampus‐dependent social recognition in mice. Hippocampus 10: 47–56, 2000. doi:10.1002/(SICI)1098-1063(2000)10:1<47::AID-HIPO5>3.0.CO;2-6. 10706216

[37] Schurz M, Radua J, Aichhorn M, Richlan F, Perner J. Fractionating theory of mind: a meta-analysis of functional brain imaging studies. Neurosci Biobehav Rev 42: 9–34, 2014. doi:10.1016/j.neubiorev.2014.01.009. 24486722

[38] Plachti A, Eickhoff SB, Hoffstaedter F, Patil KR, Laird AR, Fox PT, Amunts K, Genon S. Multimodal parcellations and extensive behavioral profiling tackling the hippocampus gradient. Cereb Cortex 29: 4595–4612, 2019. doi:10.1093/cercor/bhy336. 30721944 PMC6917521

[39] Wisse LE, Daugherty AM, Olsen RK, Berron D, Carr VA, Stark CE, et al. A harmonized segmentation protocol for hippocampal and parahippocampal subregions: why do we need one and what are the key goals? Hippocampus 27: 3–11, 2017. doi:10.1002/hipo.22671. 27862600 PMC5167633

[40] Iglesias JE, Augustinack JC, Nguyen K, Player CM, Player A, Wright M, Roy N, Frosch MP, McKee AC, Wald LL, Fischl B, Van Leemput K; Alzheimer’s Disease Neuroimaging Initiative. A computational atlas of the hippocampal formation using ex vivo, ultra-high resolution MRI: application to adaptive segmentation of in vivo MRI. Neuroimage 115: 117–137, 2015. doi:10.1016/j.neuroimage.2015.04.042. 25936807 PMC4461537

[41] Alfaro-Almagro F, Jenkinson M, Bangerter NK, Andersson JL, Griffanti L, Douaud G, Sotiropoulos SN, Jbabdi S, Hernandez-Fernandez M, Vallee E, Vidaurre D, Webster M, McCarthy P, Rorden C, Daducci A, Alexander DC, Zhang H, Dragonu I, Matthews PM, Miller KL, Smith SM. Image processing and quality control for the first 10,000 brain imaging datasets from UK Biobank. Neuroimage 166: 400–424, 2018. doi:10.1016/j.neuroimage.2017.10.034. 29079522 PMC5770339

[42] Cohen S, Hoberman HM. Positive events and social supports as buffers of life change stress. J Appl Social Pyschol 13: 99–125, 1983. doi:10.1111/j.1559-1816.1983.tb02325.x.

[43] Hawkley LC, Browne MW, Cacioppo JT. How can I connect with thee? Let me count the ways. Psychol Sci 16: 798–804, 2005. doi:10.1111/j.1467-9280.2005.01617.x. 16181443

[44] Cacioppo JT, Cacioppo S. Social relationships and health: the toxic effects of perceived social isolation. Soc Personal Psychol Compass 8: 58–72, 2014. doi:10.1111/spc3.12087. 24839458 PMC4021390

[45] Cyranowski JM, Zill N, Bode R, Butt Z, Kelly MA, Pilkonis PA, Salsman JM, Cella D. Assessing social support, companionship, and distress: National Institute of Health (NIH) Toolbox Adult Social Relationship Scales. Health Psychol 32: 293–301, 2013. doi:10.1037/a0028586. 23437856 PMC3759525

[46] Atroszko P, Pianka L, Raczyńska A, Sęktas M, Atroszko B. Validity and reliability of single-item self-report measures of social support. In: Proceedings/Research Track of the 4th Biannual CER Comparative European Research Conference International Scientific Conference. London: SCIEMCEE Publishing, 2015.

[47] Dollinger SJ, Malmquist D. Reliability and validity of single-item self-reports: with special relevance to college students’ alcohol use, religiosity, study, and social life. J Gen Psychol 136: 231–241, 2009. doi:10.3200/GENP.136.3.231-242. 19650519

[48] Mashek D, Cannaday LW, Tangney JP. Inclusion of community in self scale: a single-item pictorial measure of community connectedness. J Commun Psychol 35: 257–275, 2007. doi:10.1002/jcop.20146.

[49] Miller KL, Alfaro-Almagro F, Bangerter NK, Thomas DL, Yacoub E, Xu J, Bartsch AJ, Jbabdi S, Sotiropoulos SN, Andersson JL, Griffanti L, Douaud G, Okell TW, Weale P, Dragonu I, Garratt S, Hudson S, Collins R, Jenkinson M, Matthews PM, Smith SM. Multimodal population brain imaging in the UK Biobank prospective epidemiological study. Nat Neurosci 19: 1523–1536, 2016. doi:10.1038/nn.4393. 27643430 PMC5086094

[50] Smith SM. Fast robust automated brain extraction. Hum Brain Mapp 17: 143–155, 2002. doi:10.1002/hbm.10062. 12391568 PMC6871816

[51] Jenkinson M, Smith S. A global optimisation method for robust affine registration of brain images. Med Image Anal 5: 143–156, 2001. doi:10.1016/S1361-8415(01)00036-6. 11516708

[52] Jenkinson M, Bannister P, Brady M, Smith S. Improved optimization for the robust and accurate linear registration and motion correction of brain images. Neuroimage 17: 825–841, 2002. doi:10.1006/nimg.2002.1132. 12377157

[53] Andersson JL, Jenkinson M, Smith S. Non-Linear Registration aka Spatial Normalisation, FMRIB Technical Report TR07JA2. Oxford, UK: FMRIB Analysis Group of the University of Oxford, 2007.

[54] Zhang Y, Brady M, Smith S. Segmentation of brain MR images through a hidden Markov random field model and the expectation-maximization algorithm. IEEE Trans Med Imaging 20: 45–57, 2001. doi:10.1109/42.906424. 11293691

[55] Smith SM, Zhang Y, Jenkinson M, Chen J, Matthews PM, Federico A, De Stefano N. Accurate, robust, and automated longitudinal and cross-sectional brain change analysis. Neuroimage 17: 479–489, 2002. doi:10.1006/nimg.2002.1040. 12482100

[56] Schaefer A, Kong R, Gordon EM, Laumann TO, Zuo XN, Holmes AJ, Eickhoff SB, Yeo BT. Local-global parcellation of the human cerebral cortex from intrinsic functional connectivity MRI. Cereb Cortex 28: 3095–3114, 2018. doi:10.1093/cercor/bhx179. 28981612 PMC6095216

[57] Schurz M, Uddin LQ, Kanske P, Lamm C, Sallet J, Bernhardt BC, Mars RB, Bzdok D. Variability in brain structure and function reflects lack of peer support. Cereb Cortex 31: 4612–4627, 2021. doi:10.1093/cercor/bhab109. 33982758 PMC8408465

[58] Kernbach JM, Yeo BT, Smallwood J, Margulies DS, Thiebaut de Schotten M, Walter H, Sabuncu MR, Holmes AJ, Gramfort A, Varoquaux G, Thirion B, Bzdok D. Subspecialization within default mode nodes characterized in 10,000 UK Biobank participants. Proc Natl Acad Sci USA 115: 12295–12300, 2018. doi:10.1073/pnas.1804876115. 30420501 PMC6275484

[59] Witten DM, Tibshirani R, Hastie T. A penalized matrix decomposition, with applications to sparse principal components and canonical correlation analysis. Biostatistics 10: 515–534, 2009. doi:10.1093/biostatistics/kxp008. 19377034 PMC2697346

[60] Wang HT, Smallwood J, Mourao-Miranda J, Xia CH, Satterthwaite TD, Bassett DS, Bzdok D. Finding the needle in high-dimensional haystack: a tutorial on canonical correlation analysis. NeuroImage 216: 116745, 2020. doi:10.1016/j.neuroimage.2020.116745. 32278095

[61] Bzdok D, Nichols TE, Smith SM. Towards algorithmic analytics for large-scale datasets. Nat Mach Intell 1: 296–306, 2019. doi:10.1038/s42256-019-0069-5. 31701088 PMC6837858

[62] Efron B, Tibshirani RJ. An Introduction to the Bootstrap. Boca Raton, FL: CRC Press, 1994.

[63] Elliott J, Bodinier B, Bond TA, Chadeau-Hyam M, Evangelou E, Moons KG, Dehghan A, Muller DC, Elliott P, Tzoulaki I. Predictive accuracy of a polygenic risk score–enhanced prediction model vs a clinical risk score for coronary artery disease. JAMA 323: 636–645, 2020. doi:10.1001/jama.2019.22241. 32068818 PMC7042853

[64] International Schizophrenia Consortium, Purcell SM, Wray NR, Stone JL, Visscher PM, O’Donovan MC, Sullivan PF, Sklar P. Common polygenic variation contributes to risk of schizophrenia and bipolar disorder. Nature 460: 748–752, 2009. doi:10.1038/nature08185. 19571811 PMC3912837

[65] Inouye M, Abraham G, Nelson CP, Wood AM, Sweeting MJ, Dudbridge F, Lai FY, Kaptoge S, Brozynska M, Wang T, Ye S, Webb TR, Rutter MK, Tzoulaki I, Patel RS, Loos RJ, Keavney B, Hemingway H, Thompson J, Watkins H, Deloukas P, Di Angelantonio E, Butterworth AS, Danesh J, Samani NJ; UK Biobank CardioMetabolic Consortium CHD Working Group. Genomic risk prediction of coronary artery disease in 480,000 adults: implications for primary prevention. J Am Coll Cardiol 72: 1883–1893, 2018. doi:10.1016/j.jacc.2018.07.079. 30309464 PMC6176870

[66] Khera AV, Chaffin M, Aragam KG, Haas ME, Roselli C, Choi SH, Natarajan P, Lander ES, Lubitz SA, Ellinor PT, Kathiresan S. Genome-wide polygenic scores for common diseases identify individuals with risk equivalent to monogenic mutations. Nat Genet 50: 1219–1224, 2018. doi:10.1038/s41588-018-0183-z. 30104762 PMC6128408

[67] Kuchenbaecker KB, McGuffog L, Barrowdale D, Lee A, Soucy P, Dennis J, Domchek SM, Robson M, Spurdle AB, Ramus SJ, Mavaddat N, Terry MB, Neuhausen SL, Schmutzler RK, Simard J, Pharoah PD, Offit K, Couch FJ, Chenevix-Trench G, Easton DF, Antoniou AC. Evaluation of polygenic risk scores for breast and ovarian cancer risk prediction in BRCA1 and BRCA2 mutation carriers. J Natl Cancer Inst 109: djw302, 2017. doi:10.1093/jnci/djw302. 28376175 PMC5408990

[68] Choi SW, Mak TS, O’Reilly PF. Tutorial: a guide to performing polygenic risk score analyses. Nat Protoc 15: 2759–2772, 2020. doi:10.1038/s41596-020-0353-1. 32709988 PMC7612115

[69] Wray NR, Lin T, Austin J, McGrath JJ, Hickie IB, Murray GK, Visscher PM. From basic science to clinical application of polygenic risk scores: a primer. JAMA Psychiatry 78: 101–109, 2021. doi:10.1001/jamapsychiatry.2020.3049. 32997097

[70] Elliott LT, Sharp K, Alfaro-Almagro F, Shi S, Miller KL, Douaud G, Marchini J, Smith SM. Genome-wide association studies of brain imaging phenotypes in UK Biobank. Nature 562: 210–216, 2018. doi:10.1038/s41586-018-0571-7. 30305740 PMC6786974

[71] Smith SM, Douaud G, Chen W, Hanayik T, Alfaro-Almagro F, Sharp K, Elliott LT. An expanded set of genome-wide association studies of brain imaging phenotypes in UK Biobank. Nature Neurosci 24: 737–745, 2021. doi:10.1038/s41593-021-00826-4. 33875891 PMC7610742

[72] Lecarpentier J, Silvestri V, Kuchenbaecker KB, Barrowdale D, Dennis J, McGuffog L, et al. Prediction of breast and prostate cancer risks in male BRCA1 and BRCA2 mutation carriers using polygenic risk scores. J Clin Oncol 35: 2240–2250, 2017. doi:10.1200/JCO.2016.69.4935. 28448241 PMC5501359

[73] Fan BJ, Bailey JC, Igo RP, Kang JH, Boumenna T, Brilliant MH, et al. Association of a primary open-angle glaucoma genetic risk score with earlier age at diagnosis. JAMA Ophthalmol 137: 1190–1194, 2019. doi:10.1001/jamaophthalmol.2019.3109. 31436842 PMC6707005

[74] Meisner A, Kundu P, Zhang YD, Lan LV, Kim S, Ghandwani D, Pal Choudhury P, Berndt SI, Freedman ND, Garcia-Closas M, Chatterjee N. Combined utility of 25 disease and risk factor polygenic risk scores for stratifying risk of all-cause mortality. Am J Hum Genet 107: 418–431, 2020. doi:10.1016/j.ajhg.2020.07.002. 32758451 PMC7477009

[75] Addis DR, Wong AT, Schacter DL. Remembering the past and imagining the future: common and distinct neural substrates during event construction and elaboration. Neuropsychologia 45: 1363–1377, 2007. doi:10.1016/j.neuropsychologia.2006.10.016. 17126370 PMC1894691

[76] Andrews-Hanna JR, Smallwood J, Spreng RN. The default network and self-generated thought: component processes, dynamic control, and clinical relevance. Ann NY Acad Sci 1316: 29–52, 2014. doi:10.1111/nyas.12360. 24502540 PMC4039623

[77] Szpunar KK, Spreng RN, Schacter DL. A taxonomy of prospection: introducing an organizational framework for future-oriented cognition. Proc Natl Acad Sci USA 111: 18414–18421, 2014. doi:10.1073/pnas.1417144111. 25416592 PMC4284580

[78] Campbell KL, Madore KP, Benoit RG, Thakral PP, Schacter DL. Increased hippocampus to ventromedial prefrontal connectivity during the construction of episodic future events. Hippocampus 28: 76–80, 2018. doi:10.1002/hipo.22812. 29116660 PMC5777865

[79] Hebscher M, Levine B, Gilboa A. The precuneus and hippocampus contribute to individual differences in the unfolding of spatial representations during episodic autobiographical memory. Neuropsychologia 110: 123–133, 2018. doi:10.1016/j.neuropsychologia.2017.03.029. 28365362

[80] Parvizi J, Braga RM, Kucyi A, Veit MJ, Pinheiro-Chagas P, Perry C, Sava-Segal C, Zeineh M, van Staalduinen EK, Henderson JM, Markert M. Altered sense of self during seizures in the posteromedial cortex. Proc Natl Acad Sci USA 118: e2100522118, 2021. doi:10.1073/pnas.2100522118. 34272280 PMC8307613

[81] Barbas H, Blatt GJ. Topographically specific hippocampal projections target functionally distinct prefrontal areas in the rhesus monkey. Hippocampus 5: 511–533, 1995. doi:10.1002/hipo.450050604. 8646279

[82] Carmichael S, Price JL. Limbic connections of the orbital and medial prefrontal cortex in macaque monkeys. J Comp Neurol 363: 615–641, 1995. doi:10.1002/cne.903630408. 8847421

[83] Poletti CE, Creswell G. Fornix system efferent projections in the squirrel monkey: an experimental degeneration study. J Comp Neurol 175: 101–128, 1977. doi:10.1002/cne.901750107. 407267

[84] Aggleton JP, Wright NF, Vann SD, Saunders RC. Medial temporal lobe projections to the retrosplenial cortex of the macaque monkey. Hippocampus 22: 1883–1900, 2012. doi:10.1002/hipo.22024. 22522494 PMC3510309

[85] McCormick C, St-Laurent M, Ty A, Valiante TA, McAndrews MP. Functional and effective hippocampal–neocortical connectivity during construction and elaboration of autobiographical memory retrieval. Cereb Cortex 25: 1297–1305, 2015. doi:10.1093/cercor/bht324. 24275829 PMC4397572

[86] Yang XF, Bossmann J, Schiffhauer B, Jordan M, Immordino-Yang MH. Intrinsic default mode network connectivity predicts spontaneous verbal descriptions of autobiographical memories during social processing. Front Psychol 3: 592, 2013. doi:10.3389/fpsyg.2012.00592. 23316178 PMC3538957

[87] Hodgetts CJ, Postans M, Warne N, Varnava A, Lawrence AD, Graham KS. Distinct contributions of the fornix and inferior longitudinal fasciculus to episodic and semantic autobiographical memory. Cortex 94: 1–14, 2017. doi:10.1016/j.cortex.2017.05.010. 28710907 PMC5576916

[88] Williams AN, Ridgeway S, Postans M, Graham KS, Lawrence AD, Hodgetts CJ. The role of the pre-commissural fornix in episodic autobiographical memory and simulation. Neuropsychologia 142: 107457, 2020. doi:10.1016/j.neuropsychologia.2020.107457. 32259556 PMC7322517

[89] Twenge JM, Catanese KR, Baumeister RF. Social exclusion and the deconstructed state: time perception, meaninglessness, lethargy, lack of emotion, and self-awareness. J Pers Soc Psychol 85: 409–423, 2003. doi:10.1037/0022-3514.85.3.409. 14498779

[90] Epley N, Akalis S, Waytz A, Cacioppo JT. Creating social connection through inferential reproduction: loneliness and perceived agency in gadgets, gods, and greyhounds. Psychol Sci 19: 114–120, 2008. doi:10.1111/j.1467-9280.2008.02056.x. 18271858

[91] Zhou X, Sedikides C, Wildschut T, Gao DG. Counteracting loneliness: on the restorative function of nostalgia. Psychol Sci 19: 1023–1029, 2008. doi:10.1111/j.1467-9280.2008.02194.x. 19000213

[92] Epley N, Waytz A, Cacioppo JT. On seeing human: a three-factor theory of anthropomorphism. Psychol Rev 114: 864–886, 2007. doi:10.1037/0033-295X.114.4.864. 17907867

[93] Wildschut T, Sedikides C, Arndt J, Routledge C. Nostalgia: content, triggers, functions. J Pers Soc Psychol 91: 975–993, 2006. doi:10.1037/0022-3514.91.5.975. 17059314

[94] Tsivilis D, Vann SD, Denby C, Roberts N, Mayes AR, Montaldi D, Aggleton JP. A disproportionate role for the fornix and mammillary bodies in recall versus recognition memory. Nat Neurosci 11: 834–842, 2008. doi:10.1038/nn.2149. 18552840

[95] Jay TM, Witter MP. Distribution of hippocampal CA1 and subicular efferents in the prefrontal cortex of the rat studied by means of anterograde transport of *Phaseolus vulgaris*‐leucoagglutinin. J Comp Neurol 313: 574–586, 1991. doi:10.1002/cne.903130404. 1783682

[96] Sun C, Yang W, Martin J, Tonegawa S. Hippocampal neurons represent events as transferable units of experience. Nat Neurosci 23: 651–663, 2020. doi:10.1038/s41593-020-0614-x. 32251386 PMC11210833

[97] Bartsch T, Döhring J, Rohr A, Jansen O, Deuschl G. CA1 neurons in the human hippocampus are critical for autobiographical memory, mental time travel, and autonoetic consciousness. Proc Natl Acad Sci USA 108: 17562–17567, 2011. doi:10.1073/pnas.1110266108. 21987814 PMC3198338

[98] Zola-Morgan S, Squire LR, Amaral DG. Human amnesia and the medial temporal region: enduring memory impairment following a bilateral lesion limited to field CA1 of the hippocampus. J Neurosci 6: 2950–2967, 1986. doi:10.1523/JNEUROSCI.06-10-02950.1986. 3760943 PMC6568782

[99] Cacioppo S, Bangee M, Balogh S, Cardenas-Iniguez C, Qualter P, Cacioppo JT. Loneliness and implicit attention to social threat: a high-performance electrical neuroimaging study. Cogn Neurosci 7: 138–159, 2016. doi:10.1080/17588928.2015.1070136. 26274315

[100] Kelley A, Domesick V. The distribution of the projection from the hippocampal formation to the nucleus accumbens in the rat: an anterograde and retrograde-horseradish peroxidase study. Neuroscience 7: 2321–2335, 1982. doi:10.1016/0306-4522(82)90198-1. 6817161

[101] Cenquizca LA, Swanson LW. Spatial organization of direct hippocampal field CA1 axonal projections to the rest of the cerebral cortex. Brain Res Rev 56: 1–26, 2007. doi:10.1016/j.brainresrev.2007.05.002. 17559940 PMC2171036

[102] Wyss JM, Van Groen T. Connections between the retrosplenial cortex and the hippocampal formation in the rat: a review. Hippocampus 2: 1–11, 1992. doi:10.1002/hipo.450020102. 1308170

[103] Kobayashi Y, Amaral DG. Macaque monkey retrosplenial cortex: II. Cortical afferents. J Comp Neurol 466: 48–79, 2003. doi:10.1002/cne.10883. 14515240

[104] Rosene DL, Van Hoesen GW. Hippocampal efferents reach widespread areas of cerebral cortex and amygdala in the rhesus monkey. Science 198: 315–317, 1977. doi:10.1126/science.410102. 410102

[105] Nitzan N, McKenzie S, Beed P, English DF, Oldani S, Tukker JJ, Buzsáki G, Schmitz D. Propagation of hippocampal ripples to the neocortex by way of a subiculum-retrosplenial pathway. Nat Commun 11: 1947, 2020. doi:10.1038/s41467-020-15787-8. 32327634 PMC7181800

[106] Vann SD, Aggleton JP, Maguire EA. What does the retrosplenial cortex do? Nat Rev Neurosci 10: 792–802, 2009. doi:10.1038/nrn2733. 19812579

[107] Morton NW, Zippi EL, Noh S, Preston AR. Semantic knowledge of famous people and places is represented in hippocampus and distinct cortical networks. J Neurosci 41: 2762–2779, 2021. doi:10.1523/JNEUROSCI.2034-19.2021. 33547163 PMC8018744

[108] Mitchell AS, Czajkowski R, Zhang N, Jeffery K, Nelson AJ. Retrosplenial cortex and its role in spatial cognition. Brain Neurosci Adv 2: 2398212818757098, 2018. doi:10.1177/2398212818757098. 30221204 PMC6095108

[109] Dohmatob E, Dumas G, Bzdok D. Dark control: the default mode network as a reinforcement learning agent. Hum Brain Mapp 41: 3318–3341, 2020. doi:10.1002/hbm.25019. 32500968 PMC7375062

[110] Smallwood J, Bernhardt BC, Leech R, Bzdok D, Jefferies E, Margulies DS. The default mode network in cognition: a topographic perspective. Nat Rev Neurosci 22: 503–513, 2021. doi:10.1038/s41583-021-00474-4. 34226715

[111] Pastalkova E, Itskov V, Amarasingham A, Buzsáki G. Internally generated cell assembly sequences in the rat hippocampus. Science 321: 1322–1327, 2008. doi:10.1126/science.1159775. 18772431 PMC2570043

[112] Buzsáki G. Hippocampal sharp waves: their origin and significance. Brain Res 398: 242–252, 1986. doi:10.1016/0006-8993(86)91483-6. 3026567

[113] Diba K, Buzsáki G. Forward and reverse hippocampal place-cell sequences during ripples. Nat Neurosci 10: 1241–1242, 2007. doi:10.1038/nn1961. 17828259 PMC2039924

[114] Buzsáki G, Lai-Wo S. L, Vanderwolf CH. Cellular bases of hippocampal EEG in the behaving rat. Brain Res Rev 6: 139–171, 1983. doi:10.1016/0165-0173(83)90037-1. 6357356

[115] Ylinen A, Bragin A, Nádasdy Z, Jandó G, Szabó I, Sik A, Buzsáki G. Sharp wave-associated high-frequency oscillation (200 Hz) in the intact hippocampus: network and intracellular mechanisms. J Neurosci 15: 30–46, 1995. doi:10.1523/JNEUROSCI.15-01-00030.1995. 7823136 PMC6578299

[116] Csicsvari J, Hirase H, Mamiya A, Buzsáki G. Ensemble patterns of hippocampal CA3-CA1 neurons during sharp wave-associated population events. Neuron 28: 585–594, 2000. doi:10.1016/s0896-6273(00)00135-5. 11144366

[117] Pfeiffer BE, Foster DJ. Hippocampal place-cell sequences depict future paths to remembered goals. Nature 497: 74–79, 2013. doi:10.1038/nature12112. 23594744 PMC3990408

[118] Gupta AS, van der Meer MA, Touretzky DS, Redish AD. Hippocampal replay is not a simple function of experience. Neuron 65: 695–705, 2010. doi:10.1016/j.neuron.2010.01.034. 20223204 PMC4460981

[119] Dragoi G, Tonegawa S. Preplay of future place cell sequences by hippocampal cellular assemblies. Nature 469: 397–401, 2011. doi:10.1038/nature09633. 21179088 PMC3104398

[120] Ólafsdóttir HF, Barry C, Saleem AB, Hassabis D, Spiers HJ. Hippocampal place cells construct reward related sequences through unexplored space. eLife 4: e06063, 2015. doi:10.7554/eLife.06063. 26112828 PMC4479790

[121] Khodagholy D, Gelinas JN, Buzsáki G. Learning-enhanced coupling between ripple oscillations in association cortices and hippocampus. Science 358: 369–372, 2017. doi:10.1126/science.aan6203. 29051381 PMC5872145

[122] Rothschild G, Eban E, Frank LM. A cortical-hippocampal-cortical loop of information processing during memory consolidation. Nat Neurosci 20: 251–259, 2017. doi:10.1038/nn.4457. 27941790 PMC5783826

[123] Kaplan R, Adhikari MH, Hindriks R, Mantini D, Murayama Y, Logothetis NK, Deco G. Hippocampal sharp-wave ripples influence selective activation of the default mode network. Curr Biol 26: 686–691, 2016. doi:10.1016/j.cub.2016.01.017. 26898464 PMC4791429

[124] Logothetis NK, Eschenko O, Murayama Y, Augath M, Steudel T, Evrard H, Besserve M, Oeltermann A. Hippocampal-cortical interaction during periods of subcortical silence. Nature 491: 547–553, 2012. doi:10.1038/nature11618. 23172213

[125] Norman Y, Yeagle EM, Khuvis S, Harel M, Mehta AD, Malach R. Hippocampal sharp-wave ripples linked to visual episodic recollection in humans. Science 365: eaax1030, 2019. doi:10.1126/science.aax1030. 31416934

[126] Sosa M, Joo HR, Frank LM. Dorsal and ventral hippocampal sharp-wave ripples activate distinct nucleus accumbens networks. Neuron 105: 725–741.e8, 2020. doi:10.1016/j.neuron.2019.11.022. 31864947 PMC7035181

[127] Ambrose RE, Pfeiffer BE, Foster DJ. Reverse replay of hippocampal place cells is uniquely modulated by changing reward. Neuron 91: 1124–1136, 2016. doi:10.1016/j.neuron.2016.07.047. 27568518 PMC6013068

[128] Singer AC, Frank LM. Rewarded outcomes enhance reactivation of experience in the hippocampus. Neuron 64: 910–921, 2009. doi:10.1016/j.neuron.2009.11.016. 20064396 PMC2807414

[129] De Lavilléon G, Lacroix MM, Rondi-Reig L, Benchenane K. Explicit memory creation during sleep demonstrates a causal role of place cells in navigation. Nat Neurosci 18: 493–495, 2015. doi:10.1038/nn.3970. 25751533

[130] Robinson NT, Descamps LA, Russell LE, Buchholz MO, Bicknell BA, Antonov GK, Lau JY, Nutbrown R, Schmidt-Hieber C, Häusser M. Targeted activation of hippocampal place cells drives Memory-Guided spatial behavior. Cell 183: 1586–1599.e10, 2020. doi:10.1016/j.cell.2020.09.061. 33159859 PMC7754708

[131] Gauthier JL, Tank DW. A dedicated population for reward coding in the hippocampus. Neuron 99: 179–193.e7, 2018. doi:10.1016/j.neuron.2018.06.008. 30008297 PMC7023678

[132] Spithoven A, Cacioppo S, Goossens L, Cacioppo J. Genetic contributions to loneliness and their relevance to the evolutionary theory of loneliness. Perspect Psychol Sci 14: 376–396, 2019. doi:10.1177/1745691618812684. 30844327

[133] Boomsma DI, Willemsen G, Dolan CV, Hawkley LC, Cacioppo JT. Genetic and environmental contributions to loneliness in adults: the Netherlands Twin Register Study. Behav Genet 35: 745–752, 2005. doi:10.1007/s10519-005-6040-8. 16273322

[134] Day FR, Ong KK, Perry JR. Elucidating the genetic basis of social interaction and isolation. Nat Commun 9: 2457, 2018. doi:10.1038/s41467-018-04930-1. 29970889 PMC6030100

[135] Gao J, Davis LK, Hart AB, Sanchez-Roige S, Han L, Cacioppo JT, Palmer AA. Genome-wide association study of loneliness demonstrates a role for common variation. Neuropsychopharmacology 42: 811–821, 2017. doi:10.1038/npp.2016.197. 27629369 PMC5312064

[136] Abdellaoui A, Chen HY, Willemsen G, Ehli EA, Davies GE, Verweij KJ, Nivard MG, de Geus EJ, Boomsma DI, Cacioppo JT. Associations between loneliness and personality are mostly driven by a genetic association with neuroticism. J Pers 87: 386–397, 2019. doi:10.1111/jopy.12397. 29752830 PMC6231981

[137] Abdellaoui A, Sanchez-Roige S, Sealock J, Treur JL, Dennis J, Fontanillas P, Elson S; 23andme Research Team, Nivard MG, Ip HF, van der Zee M, Baselmans BM, Hottenga JJ, Willemsen G, Mosing M, Lu Y, Pedersen NL, Denys D, Amin N, van Duijn CM, Szilagyi I, Tiemeier H, Neumann A, Verweij KJ, Cacioppo S, Cacioppo JT, Davis LK, Palmer AA, Boomsma DI. Phenome-wide investigation of health outcomes associated with genetic predisposition to loneliness. Hum Mol Genet 28: 3853–3865, 2019. doi:10.1093/hmg/ddz219. 31518406 PMC6935385

[138] Baumeister RF, DeWall CN, Ciarocco NJ, Twenge JM. Social exclusion impairs self-regulation. J Pers Soc Psychol 88: 589–604, 2005. doi:10.1037/0022-3514.88.4.589. 15796662

[139] Hawkley LC, Thisted RA, Cacioppo JT. Loneliness predicts reduced physical activity: cross-sectional & longitudinal analyses. Health Psychol 28: 354–363, 2009. doi:10.1037/a0014400. 19450042 PMC2791498

[140] Layden EA, Cacioppo JT, Cacioppo S, Cappa SF, Dodich A, Falini A, Canessa N. Perceived social isolation is associated with altered functional connectivity in neural networks associated with tonic alertness and executive control. Neuroimage 145: 58–73, 2017. doi:10.1016/j.neuroimage.2016.09.050. 27664824

[141] Tomova L, Wang KL, Thompson T, Matthews GA, Takahashi A, Tye KM, Saxe R. Acute social isolation evokes midbrain craving responses similar to hunger. Nat Neurosci 23: 1597–1605, 2020. doi:10.1038/s41593-020-00742-z. 33230328 PMC8580014

[142] Nummenmaa L, Manninen S, Tuominen L, Hirvonen J, Kalliokoski KK, Nuutila P, Jääskeläinen IP, Hari R, Dunbar RI, Sams M. Adult attachment style is associated with cerebral μ‐opioid receptor availability in humans. Hum Brain Mapp 36: 3621–3628, 2015. doi:10.1002/hbm.22866. 26046928 PMC6869236

[143] Hertenstein MJ, Verkamp JM, Kerestes AM, Holmes RM. The communicative functions of touch in humans, nonhuman primates, and rats: a review and synthesis of the empirical research. Genet Soc Gen Psychol Monogr 132: 5–94, 2006. doi:10.3200/mono.132.1.5-94. 17345871

[144] Nummenmaa L, Tuominen L, Dunbar R, Hirvonen J, Manninen S, Arponen E, Machin A, Hari R, Jääskeläinen IP, Sams M. Social touch modulates endogenous μ-opioid system activity in humans. NeuroImage 138: 242–247, 2016. doi:10.1016/j.neuroimage.2016.05.063. 27238727

[145] Belsky J, Bakermans-Kranenburg MJ, Van IJzendoorn MH. For better and for worse: differential susceptibility to environmental influences. Cur Dir Psychol Sci 16: 300–304, 2007. doi:10.1111/j.1467-8721.2007.00525.x.

[146] Heinrichs M, Baumgartner T, Kirschbaum C, Ehlert U. Social support and oxytocin interact to suppress cortisol and subjective responses to psychosocial stress. Biol Psychiatry 54: 1389–1398, 2003. doi:10.1016/s0006-3223(03)00465-7. 14675803

[147] Chen FS, Kumsta R, Von Dawans B, Monakhov M, Ebstein RP, Heinrichs M. Common oxytocin receptor gene (*OXTR*) polymorphism and social support interact to reduce stress in humans. Proc Natl Acad Sci USA 108: 19937–19942, 2011. doi:10.1073/pnas.1113079108. 22123970 PMC3250137

[148] Zayan R. The specificity of social stress. Behav Processes 25: 81–93, 1991. doi:10.1016/0376-6357(91)90011-N. 24923968

[149] Sapolsky RM. Why stress is bad for your brain. Science 273: 749–750, 1996. doi:10.1126/science.273.5276.749. 8701325

[150] Conrad CD, Ortiz JB, Judd JM. Chronic stress and hippocampal dendritic complexity: methodological and functional considerations. Physiol Behav 178: 66–81, 2017. doi:10.1016/j.physbeh.2016.11.017. 27887995

[151] Magariños AM, McEwen BS. Stress-induced atrophy of apical dendrites of hippocampal CA3c neurons: comparison of stressors. Neuroscience 69: 83–88, 1995. doi:10.1016/0306-4522(95)00256-I. 8637635

[152] Magariños AM, Verdugo JM, McEwen BS. Chronic stress alters synaptic terminal structure in hippocampus. Proc Natl Acad Sci USA 94: 14002–14008, 1997. doi:10.1073/pnas.94.25.14002. 9391142 PMC28422

[153] Magariños AM, McEwen BS, Flügge G, Fuchs E. Chronic psychosocial stress causes apical dendritic atrophy of hippocampal CA3 pyramidal neurons in subordinate tree shrews. J Neurosci 16: 3534–3540, 1996. doi:10.1523/JNEUROSCI.16-10-03534.1996. 8627386 PMC6579123

[154] Watanabe Y, Gould E, McEwen BS. Stress induces atrophy of apical dendrites of hippocampal CA3 pyramidal neurons. Brain Res 588: 341–345, 1992. doi:10.1016/0006-8993(92)91597-8. 1393587

[155] Woolley CS, Gould E, McEwen BS. Exposure to excess glucocorticoids alters dendritic morphology of adult hippocampal pyramidal neurons. Brain Res 531: 225–231, 1990. doi:10.1016/0006-8993(90)90778-a. 1705153

[156] Alfarez D, Karst H, Velzing E, Joëls M, Krugers HJ. Opposite effects of glucocorticoid receptor activation on hippocampal CA1 dendritic complexity in chronically stressed and handled animals. Hippocampus 18: 20–28, 2008 [Erratum in *Hippocampus* 18: 238, 2008]. doi:10.1002/hipo.20360. 17708551

[157] Maras PM, Molet J, Chen Y, Rice C, Ji SG, Solodkin A, Baram TZ. Preferential loss of dorsal-hippocampus synapses underlies memory impairments provoked by short, multimodal stress. Mol Psychiatry 19: 811–822, 2014. doi:10.1038/mp.2014.12. 24589888 PMC4074447

[158] Cole SW. Social regulation of leukocyte homeostasis: the role of glucocorticoid sensitivity. Brain Behav Immun 22: 1049–1055, 2008. doi:10.1016/j.bbi.2008.02.006. 18394861 PMC3004947

[159] Adam EK, Hawkley LC, Kudielka BM, Cacioppo JT. Day-to-day dynamics of experience–cortisol associations in a population-based sample of older adults. Proc Natl Acad Sci USA 103: 17058–17063, 2006. doi:10.1073/pnas.0605053103. 17075058 PMC1636578

[160] Steptoe A, Owen N, Kunz-Ebrecht SR, Brydon L. Loneliness and neuroendocrine, cardiovascular, and inflammatory stress responses in middle-aged men and women. Psychoneuroendocrinology 29: 593–611, 2004. doi:10.1016/S0306-4530(03)00086-6. 15041083

[161] Xia N, Li H. Loneliness, social isolation, and cardiovascular health. Antioxid Redox Signal 28: 837–851, 2018. doi:10.1089/ars.2017.7312. 28903579 PMC5831910

[162] Cerqueira JJ, Mailliet F, Almeida OF, Jay TM, Sousa N. The prefrontal cortex as a key target of the maladaptive response to stress. J Neurosci 27: 2781–2787, 2007. doi:10.1523/JNEUROSCI.4372-06.2007. 17360899 PMC6672565

[163] Yuan R, Nechvatal JM, Buckmaster CL, Ayash S, Parker KJ, Schatzberg AF, Lyons DM, Menon V. Long-term effects of intermittent early life stress on primate prefrontal-subcortical functional connectivity. Neuropsychopharmacology 46: 1348–1356, 2021. doi:10.1038/s41386-021-00956-0. 33495547 PMC8134590

[164] McEwen BS, Magarinos AM. Stress effects on morphology and function of the hippocampus. Ann NY Acad Sci 821: 271–284, 1997. doi:10.1111/j.1749-6632.1997.tb48286.x. 9238211

[165] Conrad CD. Chronic stress-induced hippocampal vulnerability: the glucocorticoid vulnerability hypothesis. Rev Neurosci 19: 395–411, 2008. 19317179 10.1515/revneuro.2008.19.6.395 PMC2746750

[166] Champagne DL, Bagot RC, van Hasselt F, Ramakers G, Meaney MJ, De Kloet ER, Joëls M, Krugers H. Maternal care and hippocampal plasticity: evidence for experience-dependent structural plasticity, altered synaptic functioning, and differential responsiveness to glucocorticoids and stress. J Neurosci 28: 6037–6045, 2008. doi:10.1523/JNEUROSCI.0526-08.2008. 18524909 PMC6670331

[167] Hutchinson KM, McLaughlin KJ, Wright RL, Ortiz JB, Anouti DP, Mika A, Diamond DM, Conrad CD. Environmental enrichment protects against the effects of chronic stress on cognitive and morphological measures of hippocampal integrity. Neurobiol Learn Mem 97: 250–260, 2012. doi:10.1016/j.nlm.2012.01.003. 22266288 PMC7051243

[168] Sandi C, Davies HA, Cordero MI, Rodriguez JJ, Popov VI, Stewart MG. Rapid reversal of stress induced loss of synapses in CA3 of rat hippocampus following water maze training. Eur J Neurosci 17: 2447–2456, 2003. doi:10.1046/j.1460-9568.2003.02675.x. 12814376

[169] Liston C, McEwen BS, Casey B. Psychosocial stress reversibly disrupts prefrontal processing and attentional control. Proc Natl Acad Sci USA 106: 912–917, 2009. doi:10.1073/pnas.0807041106. 19139412 PMC2621252

[170] Soares JM, Sampaio A, Ferreira LM, Santos N, Marques F, Palha JA, Cerqueira J, Sousa N. Stress-induced changes in human decision-making are reversible. Transl Psychiatry 2: e131, 2012. doi:10.1038/tp.2012.59. 22760555 PMC3410630

[171] Radley JJ, Rocher AB, Janssen WG, Hof PR, McEwen BS, Morrison JH. Reversibility of apical dendritic retraction in the rat medial prefrontal cortex following repeated stress. Exp Neurol 196: 199–203, 2005. doi:10.1016/j.expneurol.2005.07.008. 16095592

[172] Bloss EB, Janssen WG, McEwen BS, Morrison JH. Interactive effects of stress and aging on structural plasticity in the prefrontal cortex. J Neurosci 30: 6726–6731, 2010. doi:10.1523/JNEUROSCI.0759-10.2010. 20463234 PMC2888496

[173] Bzdok D, Floris DL, Marquand AF. Analysing brain networks in population neuroscience: a case for the Bayesian philosophy. Philos Trans R Soc Lond B Biol Sci 375: 20190661, 2020. doi:10.1098/rstb.2019.0661. 32089111 PMC7061951

[174] Bzdok D, Yeo BTT. Inference in the age of big data: future perspectives on neuroscience. Neuroimage 155: 549–564, 2017. doi:10.1016/j.neuroimage.2017.04.061. 28456584

